# Walras modulates sex-dependent endoplasmic reticulum stress in cardiomyopathy

**DOI:** 10.3389/fphys.2026.1740128

**Published:** 2026-05-11

**Authors:** Francisco J. Martinez-Amaro, Carlos Garcia-Padilla, Fernando Bonet, Elena Alonso-Villa, Rocio Toro, Houria Daimi, Eva Vargas, Ivan Hernandez, Borja Vilaplana-Martí, Ignacio Perez de Castro, Laetitia Bouchard, Fabien Hubert, Francesca Rochais, Antoine Muchir, Michaela Veliova, José Antonio Enriquez, Alejandro Salguero, Jose Luis De La Pompa, Estefania Lozano-Velasco, Diego Franco

**Affiliations:** 1 Cardiovascular Research Group, Department of Experimental Biology, University of Jaen, Jaen, Spain; 2 Medina Foundation, Technology Park of Health Sciences, Granada, Spain; 3 Department of Medicine, School of Medicine, University of Cádiz (UCA), Cádiz, Spain; 4 Research Unit, Biomedical Research and Innovation Institute of Cadiz (INiBICA), Puerta del Mar University Hospital, Cadiz, Spain; 5 Institute of Applied Molecular Medicine (IMMA), Department of Basis Medical Sciences, Facultad de Medicina, Universidad San Pablo-CEU, CEU Universities, Madrid, Spain; 6 Laboratory of Human Genome and Multifactorial Diseases (LR12ES07), Faculty of Pharmacy, University of Monastir, Monastir, Tunisia; 7 Systems Biology Unit, Department of Experimental Biology, University of Jaen, Jaen, Spain; 8 Instituto de Salud Carlos III, Madrid, Spain; 9 Aix-Marseille Univ, MMG, Inserm U1251, Marseille, France; 10 Sorbonne Université, INSERM U974, Institute of Myology, Center of Research in Myology, Paris, France; 11 Centro Nacional de Investigaciones Cardiovasculares Carlos III, Madrid, Spain; 12 CIBER de Fragilidad y Envejecimiento Saludable (CIBERFES), Madrid, Spain; 13 Intercellular Signaling in Cardiovascular Development and Disease Laboratory, Centro Nacional de Investigaciones Cardiovasculares Carlos III, Madrid, Spain; 14 Ciber Cardiovascular (CiberCV), Madrid, Spain

**Keywords:** dilated cardiomyopathy, ERS, hypertrophic cardiomyopathy, lncRNAs, *Walras*, UPR

## Abstract

**Introduction:**

Cardiovascular diseases (CVDs) are the leading cause of death globally, taking an estimated 17.9 million lives each year. Most heart cardiomyopathies result in an increased need for protein production that translates into an increased endoplasmic reticulum stress and therefore in the activation of the unfolded protein response pathway (UPR). The sustained activation of this pathway produces cell death and worsens the course of the disease. The role of lncRNAs in UPR signalling and their impact in several cardiomyopathies is beginning to be addressed.

**Methods:**

To conduct our study we have performed real time PCR (qPCR), immunochemistry (IMQ), SeaHorse mithocondrial activity, Western blot (WB), Mass spectrometry (MS) and cell viability analysis.

**Results:**

Our results demonstrate a sex-dependent regulation of *Walar*, *Walaa*, *Wallrd*, *Walrad* and *Walras* lncRNAs in different dilated cardiomyopathy (DCM) and hypertrophic cardiomyopathy (HCM) murine experimental models. Functional assays demonstrated that Walras overexpression leads to *unfolded protein response* (UPR) pathway activation and increased apoptosis, and additionally it also impairs mitochondrial function. Mechanistically, *Walras* physically interacts with calumenin (*CALU*), repressing its protein levels by promoting proteosomal degradation. Finally, we proved that *APO02340.1*, a *Walras* human homologue exerts a similar role.

**Discussion:**

Our data demonstrate that *Walras* and *APO02340.1* modulate UPR associated apoptosis by regulating *CALU* protein turnover and thus acting as deletereous factors in several cardiomyophaties.

## 1 Introduction

Dilated cardiomyopathy (DCM) is characterized by an enlarged and dysfunctional left ventricle that encompasses various etiological causes. Globally, this condition is the most common cause of heart failure (HF) and heart transplantation (Schultheiss et al., 2019). Interestingly, several studies have recently pinpointed that DCM is more frequent in men (60–77%); having lower left ventricle ejection fraction (LVEF), worse outcomes and higher mortality rates as compared to women (Cannata et al., 2020). Furthermore, large scale genome-wide association studies have demonstrated the existence of risk SNPs that are associated with men who have suffered DCM, but are not present in women with this disease, suggesting a genetic predisposition associated with sex (Fairweather et al., 2023; Zhang et al., 2023).

It is important to highlight that the majority of deregulated genes associated to DCM are involved in forming the cardiomyocyte cytoskeleton, the sarcomere structure, the nuclear envelope, and/or the regulation of calcium/mitochondrial homeostasis (Bonet et al., 2024). Nevertheless, the cellular and molecular mechanisms underlying DCM are largely unknown. In this context, the role of endoplasmic reticulum stress (ERS) in the pathogenesis and progression of DCM has been discussed for decades (Martinez-Amaro et al., 2023). An extensive number of factors can disrupt normal endoplasmic reticulum (ER) function and cause ERS, including hypoxia, hyperglycemia, oxidative stress, lipotoxicity or inflammation, respectively (Zhang et al., 2017; Zhao et al., 2023; An et al., 2024). In addition, apoptosis plays a crucial role in myocardial injury, and cardiomyocyte homeostasis involves several cellular processes, including ERS and autophagy (Dong et al., 2019; Fang et al., 2021). Curiously, ERS signaling has cytoprotective effects and is required to maintain cardiomyocyte function and homeostasis (Jiang et al., 2021). However, if ERS is prolonged, cardiomyocyte dysfunction and apoptosis are triggered. Importantly, increased ERS results in the presence of autophagic vesicles in the left ventricle of patients with DCM (Sánchez-Esteban et al., 2021), further supporting the role of ERS in DCM pathophysiology.

Additionally, prolonged ERS leads to the activation of pathways that eliminate defective cells, as occurs in DCM, increasing cardiomyocyte apoptosis (Chen et al., 2024). ERS activates the unfolded protein response (UPR) which is mediated by three ER stress sensors, Inositol requiring enzyme *(*IRE1α), Protein kinase R-like endoplasmic reticulum kinase *(*PERK) and Activating Transcription Factor 6 (ATF6). Upon stress, Binding immunoglobulin Protein (BiP) dissociation allows IRE1α to dimerize and activate its kinase/RNase domains. Active IRE1α then splices X-box binding protein 1 (XBP1) mRNA into XBP1s (a transcription factor upregulating chaperones and ERAD genes) and also degrades select ER-targeted transcripts (RIDD). In parallel, PERK oligomerizes and phosphorylates eIF2α, which dampens global translation while enabling activating transcription factor 4 (ATF4) translation; ATF4 in turn induces stress-adaptive genes including CCAAT/enhancer-binding protein homologous protein (CHOP), a pro-apoptotic factor). Simultaneously, ATF6 moves to the Golgi and is cleaved by S1P/S2P, releasing the ATF6(N) fragment that enters the nucleus to upregulate ER chaperones (e.g. BiP) and UPR genes (such as XBP1). These branches exhibit crosstalk – for example, the PERK/ATF4 axis can amplify IRE1–XBP1 signaling – to fine-tune the response. Collectively, IRE1, PERK and ATF6 increase folding capacity and clearance of misfolded proteins to reestablish ER function, whereas unresolved stress activates CHOP/JNK pathways leading to apoptosis (Martinez-Amaro et al., 2023).

It has also been shown that plasma from patients with ischemic dilated cardiomyopathy (ICM) is enriched in miR-16-5p and promotes ER stress-induced apoptosis in cardiomyocytes *in vitro* (Calderon-Dominguez et al., 2021). Moreover, the administration of astaxanthin (AST) ameliorates ethanol-induced DCM by inhibiting cardiac ERS and the resulting apoptosis, and this involves Heat Shock Protein Family A (Hsp70) Member 5 (GRP78) (Wang et al., 2021). Finally, mutations in F-box protein 32 (FBXO32) cause DCM via upregulation of ERS-associated apoptosis (Al-Yacoub et al., 2021). These findings indicate that ERS-induced apoptosis promotes the development of DCM. Therefore, the relationship between ERS and apoptosis in DCM requires further investigation, and may represent a therapeutic target for DCM.

The role of lncRNAs in the modulation of several ERS pathways on cardiac disease context have been widely described, i.e – Maternally Expressed 3 (MEG3) and Differentiation Antagonizing Non-Protein Coding RNA (DANCR) in myocardial infarction (MI), H19 Imprinted Maternally Expressed Transcript in diabetic cardiomyophaty or HypERlnc in atherosclerosis (Bischoff et al., 2017; Li et al., 2019; Li et al., 2021; Wang et al., 2022). Curiously, the mayority of them are described as protective agents against UPR associated apoptosis. Recently, Qiu et al. (2021) reported a severe downregulation of AC061961.2, an unknown annotated lncRNA, in heart samples from DCM patients. Functional analysis of this lncRNA demonstrated that AC061961.2 overexpression resulted in lower apoptosis associated to adriamycin-induced DCM, by repressing GRP78, CHOP and caspase 3 (CASP3) protein expression levels and increasing Blc2 antiapoptotic factor expression (Qiu et al., 2021).

We have previously described a subset of lncRNAs associated to Wnt signaling - *Walar, Walaa, Walrad, Wallrd* and *Walras*- in the context of atrial fibrillation (AF). Functional assays demonstrated that *Walras* modulates the expression of several cytoskeleton proteins such as Myosin Heavy Chain 9 (MYH9), actinin alpha 1 (ACTN1) and actinin alpha 4 (ACTN4), contributing to the cardiomyocyte architecture and cell-cell adhesion. Furthermore, two human *Walras* homologues were identified– LINC02740 and AP002340.1-, both of which are also deregulated in atrial samples of AF human patients, suggesting a plausible contribution to AF pathophysiology (García-Padilla et al., 2022). Importantly, AF is the most common arrhythmia in patients with DCM and both diseases share several molecular pathways (Aleksova et al., 2010; Buckley et al., 2021; Gan et al., 2023). Furthermore, AF is also more prevalent in men than in woman (Ko et al., 2016; Westerman and Wenger, 2019).

In this study we aim to dissect the functional role of lncRNAs associated to Wnt signaling described above in the pathogenesis of cardiomyopathies and their relationship with ERS and UPR signaling pathways. Our results demonstrate a sex-dependent regulation of these lncRNAs in different DCM and hypertrophic cardiomyopathy (HCM) murine models, further supported by *in vitro* analysis with β-estradiol. Functional assays demonstrated that *Walras* overexpression leads to UPR pathway activation and increased apoptosis. Additionally, *Walras* overexpression also impairs mitochondrial function. Mechanistically, *Walras* physically interacts with calumenin, repressing its protein levels by promoting proteosomal degradation. Finally, we prove that APO02340.1, a *Walras* human homologue, exerts a similar role, activating the UPR pathway and increasing apoptosis.

## 2 Materials and methods

### 2.1 Mouse lines and mouse tissue samples

Three distinct genetically engineered mouse models were used in this study. *αMHC^MerCreMer^ Lmna^F/F 94^*, *Lmna^R249W^* ^95^ and *Mybc3-p109* (Pau, Salguero et al., in preparation). *αMHC^MerCreMer^*; *Lmna^F/F^* (mouse line courtesy from Antoine Muchir and Francesca Rochais) (Hubert et al., 2022) and Lmna^R249W 95^ developed dilated cardiomyopathy while Mybc3-p109 developed hypertrophic cardiomyopathy. Floxed allele recombination using inducible CRE in *αMHC^MerCreMer^*; *Lmna^F/F^* mouse was achieved by intraperitoneal tamoxifen injection in adult mice at a dose of 1mg/20g. Tissue samples from these three distinct models, i.e. dilated cardiomyopathy (*Lmna^R249W^*, *αMHC^MerCreMer^*; *Lmna^F/F^*) and hypertrophic cardiomyopathy (*Mybc3-p109*) were collected from their corresponding laboratories. Experimental acute myocardial infarction was performed in wildtype mice in collaboration with Francesca Rochais (MMG, Aix-Marseille Univ, France) as previously described (Hubert et al., 2022).

Experiments using animals were performed under protocols approved by the ISCIII Ethical Committee (Madrid, Spain; protocol PROEX 164-18), Departmental Direction of Veterinary Services of the French Ministry of Agriculture and the local ethics committee (APAFIS19069–2019021113531586 v3; APAFIS #26541–2020070912206899 v1) and by the Universidad de Jaén Ethical Committee (Jaén Spain; protocol 18/11/14/155) in accordance with Spanish Royal Decree 53/2013, European Directive 2010/63/EU and other relevant guidelines. Pregnant females, male and neonatal mice were euthanized by cervical dislocation and by decapitation, respectively. This study is reported in accordance with ARRIVE guidelines.

### 2.2 Mouse heart function analyses (ecocardiography)

Mouse heart function was evaluated at the CERIMED-Marseille, by transthoracic echocardiography using a Vevo 2100 VisualSonics. Mice were anaesthetized with isoflurane in oxygen (2% for induction and 1% for maintenance) and placed on a warm pad at the supine position. All echocardiography measurements were performed in a blinded manner. An extended protocol of echocardiography has been described in **Supplementary Table 4**.

### 2.3 *Walras*, *APO02340.1* and *LINC02761* plasmids

The *Walras*, *APO02340.1* and *LINC02761* full-length sequences were PCR amplified from mouse and human cDNAs, respectively and subsequently inserted into pcDNA3.1(+) IRES GFP (Addgene #51406). A plasmid carrying a non-targeting sequence (pcDNA3.1(+) IRES-GFP) was used as a negative control. The primer sequences of pcDNA3.1(+) IRES GFP *Walras*, *AP002340.1* and *LINC02761* were as follows: *Walras*_BamHI-F: GGATCCTGCTACAATGGAGGGCTTCAG; *Walras*_EcoRI-R: GAATTCCACCTATAACCAGCCACTTTC; *APO02340.1*_BamHI-F: GGATCCAGACAGGATCTCATCATCTTGCACACAC; *AP002340.1*_EcoRI-R: GAATTCCTCCAGACCTGCGTCCTACAGAA; *LINC02761*_BamHI-F: GGATCCGTTGGTGGCTGCATCCCAATG; *LINC02761*_EcoRI-R: GAATTCTCACTACAGCTGGTGTTTACTAAGCGT.

### 2.4 Cell culture treatments

HL1 cell line was cultured in Claycomb medium supplemented with 10% Fetal Bovine serum (FBS), 100U/ml penicillin/streptomycin, 200 nM of L-glutamine and Norepinephrine 0,1mM as previously reported (Muñoz-Gallardo et al., 2024). AC16 Human cardiomyocyte cell line was cultured DMEM F12, supplemented with FBS 12,5%, 100U/ml penicillin/streptomycin and 200 nM of L-glutamine as previously reported (Alonso-Villa et al., 2024). Both lines were cultured in 60 cm (Cannata et al., 2020) culture disks at 37 °C in a humidified atmosphere of 5% CO2. Sub-cultured cells were treated with *Walras* siRNA (Sigma, Aldrich, Munich, Germany), and pcDNA3.1(+) IRES-GFP-*Walras*, *-LINC02761* or -*APO02340.1* plasmids, respectively, using Lipofectamine 2000 (Invitrogen) following the manufacturer’s guidelines. HL1 and AC16 cell lines were treated with β-estradiol (20 nm), testosterone (50 μm) and/or tunicamycin (2 µm) during 24 hours, respectively.

### 2.5 Adenovirus construction and infection

*Walras* full-length was cloned in pAV-CMV promoter vector (pAV-CMV-Walras-GFP #VB230904-1200fkw) (VectorBuilding, Guangzhou, China). An adenovirus carrying a scramble sequence (pAV-CMV-GFP) was used as a negative control (VectorBuilding, Guangzhou, China). HL1 cells were infected using a pAV-CMV-*Walras*-GFP or pAV-CMV-GFP, respectively, at 240 M.O.I during 16 hours in a 96 well plate precoated with gelatin/fibronectin with a complete HL1 Claycomb medium. Afterwards, the cells were refreshed with Claycomb medium in the absence of infectious particles and processed for subsequent analysis.

### 2.6 Cell and/or tissue samples

Whole ventricles from *to DCM and HCM murine models* were used to this study. MI samples from left ventricles were dissected in three different areas – infarct zone (IZ), border zone (BZ) and remote zone (RZ), according to the proximity to infarct region. Tissue samples were collected and frozen at 80 °C until analyzed. Likewise, treated and control cells were collected using Trypsin 0.25% EDTA by centrifugation and frozen at 80 °C until analyzed.

### 2.7 RNA isolation

RNA from treated cells were isolated using Reliaprep Rna Miniprep System (Promega) following manufacture’s protocol. In all cases at least three different pools of each condition were collected from different culture wells. RNA from heart tissues were extracted and purified using Trizol reactive (Invitrogen) following manufacture recommendations. RNA isolated was storaged at -80°C until used.

### 2.8 cDNA synthesis and qPCR analysis

RNA retrotranscription was performed using Maxima first strand cDNA synthesis (Thermo Scientific). To perform real time qPCR, we used the corresponding cDNA, primers and SYBR Green Mix. Reactions were performed in 10 μL total volume, using 96-wells or 384-well plates and optical sealing tape in a CFX96TM or CFX384TM thermocycler (Bio-Rad), respectively. Amplification conditions were denaturalization step of 95°C for 10 min, followed by 45 cycles of 95°C for 5s, 60°C for 10s, 75°C for 7s, with final steps of 95°C for 10s. To determine the relative expression of the different genes, *Gadph* was used as normalization control. Each qPCR reaction was performed by triplicate and repeated three times to obtain representative means. Primers were designed using primer3 software and validated using Blast software. No amplification was observed in PCR control reactions with only water as template. The Livak & Schmittgen (Livak and Schmittgen, 2001) method for relative qPCR analyses was used and normalized to control (wild-type) values. Primers sequence is described in **Supplementary Table 1**.

### 2.7 Nuclear/cytoplasmic distribution

Nuclear and cytoplasmic RNA fractions from HL1 and AC16 cells were isolated with Cytoplasmic & Nuclear RNA Purification Kit (Norgen, Belmont, CA, USA), respectively, according with manufacture’s recommendation. RT-qPCR analysis for nuclear enriched *Rpb1* mRNA marker and cytoplasmic *Gapdh* mRNA marker were performed to validate enrichment on each subcellular fraction.

### 2.9 Cell viability assay

Cell viability of HL1 and AC16 exposed to different experimental conditions was evaluated using Apoptosis/Necrosis Detection Kit (Blue, Red, Green) ab176750 (Abcam) following the manufacter’s intructions.

### 2.10 LncRNA pulldown assays and mass spectrometry

Biotinylated RNA from *LINC02761* and *APO02340.1* genes was synthesized from AC16 cDNA using forward primers containing T7 RNA polymerase promoter sequence (**Table 1**), respectively. DNA templates were purified using mi-PCR purification kit (Metabion) and MaxiScript T7 kit (Ambion) was used to synthesize biotinylated transcripts. Whole-cell lysate from AC16 cells (500 μg) were incubated with 1 μg of biotinylated RNA for 2 h at room temperature. Using Streptavidin-coupled Dynabeads (Invitrogen) the complexes formed were isolated as previously described Panda et al. (2016) and analyzed by mass spectrometry (MS) (Panda et al., 2016).

**Table 1:** Primers sequences.

Gene	Sequence	Specie
Walras/Gm44934 F	GCTACAATGGAGGGCTTCAG	Mus musculus
Walras/Gm44934 R	CAGCTTCTTCATGGCCTCAT	Mus musculus
Gm26538 F	ACASGCAAAGGATTGGTGAGC	Mus musculus
Gm26538 R	TTACAGAAGAGCCTGCCACA	Mus musculus
Walaa/Gm 45188 F	ACAGAGAATACGGGCACACC	Mus musculus
Walaa/Gm 45188 R	TCAGAGGAGCTTCCGAGAAA	Mus musculus
Walrad/Gm44653 F	GGTTCTGAAGAGGGCAAAGA	Mus musculus
Walrad/Gm44653 R	CCACTACCATAGGGGCTGTG	Mus musculus
Wallrd/2010110K18Rik F	GCTGAGACCGATGAAGTGGT	Mus musculus
Wallrd/2010110K18Rik R	CATCCTTGTGGCTGCCTACA	Mus musculus
ATF6 F	TACCACCCACAACAAGACCA	Mus musculus
ATF6 R	TGATGATCCCGGAGATAAGG	Mus musculus
IRE1 F	CGAATAGAAAAGGAGGCCTTG	Mus musculus
IRE1 R	CTCGGAGGAGGTCTCTCACA	Mus musculus
PERK F	TTCATGGAAACAACTACTCCCATA	Mus musculus
PERK R	TGGGGATATTTCTGAGTGAACA	Mus musculus
BIP F	CAGATCTTCTCCACGGCTTC	Mus musculus
BIP R	TTCAGCTGTCACTCGGAGAA	Mus musculus
ATF4 F	GAAACCTCATGGGTTCTCCA	Mus musculus
ATF4 R	AGAGCTCATCTGGCATGGTT	Mus musculus
Bcl-2 F	AGTACCTGAACCGGCATCTG	Mus musculus
Bcl-2 R	CAGGTATGCACCCAGAGTGA	Mus musculus

Peptide mixtures were loaded into a peptide trap cartridge and eluted into a reversed-phase PicoFrit column (New Objective, Woburn, MA, USA). These peptides were sprayed and ionized, using a Nanospray Flex Ion Source ES071 (Thermo Scientific), into the mass spectrometer (Thermo Scientific Q-Exactive hybrid Quadrupole-Orbitrap Mass Spectrometer with Thermo Dionex UltiMate 3000 RSLCnano System). Using the Thermo Proteome Discoverer 1.4.1 platform, proteins were identified. Database search against public mouse and human protein database from NCBI was performed through the Proteome Discoverer 1.4.1 platform (Orsburn, 2021), respectively.

### 2.11 Interaction and functional enrichment analyses

Protein-protein interaction (PPI) analyses were performed using STRING platform (version 12.0) (Szklarczyk et al., 2023) to assess the network of interactions among the target proteins. The minimum required interaction score was set to the highest level (0.9). Further, functional enrichment analysis was conducted using the Database for Annotation, Visualization and Integrated Discovery (DAVID) tool (Sherman et al., 2022) in order to elucidate the Gene Ontology (GO) terms and pathways associated with our dataset. The top significant Biological Processes (BP), Molecular Functions (MF) and Cellular Components (CC) were detected for each set of genes.

### 2.12 RNA inmunoprecipation assay

For immunoprecipitation of endogenous calumenin, HL1 and AC16 cells were lysated in PEB buffer for 10 min on ice and centrifuged at 10–000 g for 15 min at 4 °C, respectively. Afterwards the supernatants were incubated with protein A Sepharose beads (Abcam) coated with antibodies that recognized calumenin (sc-271357- Santacruz) or control IgG (Abcam) for 2 h at 4 °C, respectively. The corresponding beads were washed with NT2 buffer (50 mM Tris–HCl [pH 7.5], 150 mM NaCl, 1 mM MgCl2, 0.05% NP-40). Protein complexes were incubated with 20 units of DNase I (15 min at 37 °C). In this step, an aliquot from each reaction was isolated for Western blot validation. Subsequently they were further incubated with 0.1% SDS/0.5 mg/ml Proteinase K (30 min at 55 °C) to remove proteins. The RNA isolated from the IP materials were further assessed by RT-qPCR analysis. This assay was performed as previously described by Garcia-Padilla et al. (2022) (García-Padilla et al., 2022).

### 2.13 Immunofluorescence analyses by confocal laser scanning microscopy

Cells were cultures in four chambered culture wells, rinsed with PBS, fixed with 4% PFA during 10 min and rinsed again twice with PBS. Afterwards, permeabilization of the cells was performed using NH_4_Cl 50 nM and Triton 100X 0,2% diluted in PBS for 10 min, followed by a PBS rinsed twice during 5 min and blocked with Gelatine 0,2% in PBS during 10 min twice. The cells were incubated with the calumenin-specific primary antibody (sc-271357- SantaCruz) diluted in blocking solution overnight at 4 °C. Subsequently, the cells were rinsed three times with PBS during 10 min and incubated with a goat anti-mouse secondary antibody (AlexaFluor 546 # A-11030, Thermo Fisher Scientific, Rockford, IL, USA) diluted in blocking solution over 30 min. Finally, the cells were rinsed four times in PBS during 10 min and kept in PBS until analyzed by confocal microscopy.

### 2.14 SeaHorse analysis

The Seahorse XFe96 Extracellular Flux Analyzer (Agilent Technologies, Santa Clara, CA, USA) was used to measure oxygen consumption rate (OCR) in HL1 cells. Cells were transfected in customized Seahorse 96-well plates and infected as described above. 24h after transfection, the cells were incubated for 1h in XF Assay Medium (Seahorse Bioscience, Santa Clara, CA, USA) plus 5 mM glucose for a typical bioenergetic profile; the OCR of basal respiration was initially measured, followed by exposure to 1 µM oligomycin (an ATP synthase inhibitor), which allowed the detection of the amount of O_2_ consumed by ATP synthesis, H+ leak, and other oxidases. Then, 1 µM of the uncoupler carbonyl cyanide 4-(trifluoromethoxy) phenylhydrazone (FCCP) was added to quantify the maximal respiratory capacity, followed by 1 µM of rotenone and 1 µM of antimycin A, a mix of inhibitors of the complexes I and III of the mitochondrial electron transport chain (ETC), which fully depleted mitochondrial O_2_ consumption. OCR data were calculated using Wave software v. 2.6.1 (Agilent Technologies, Santa Clara, CA, USA), and data were normalized per nuclear staining signal. OCR measures were obtained from 4 technic replicates from each condition. ad-Walras condition was compared to ad-GFP condition while Tunycamicine condiction was compared to control sample.

### 2.15 Western blot

Transfected HL1 cells were harvested in RIPA buffer, and protein concentration was determined using a Pierce BCA protein assay kit (Thermo Fisher Scientific, Rockford, IL, USA). Equal amounts (20 µg) of protein were loaded, electrophoresed on 8–12% SDS-PAGE. BSA blocking (2%) was used by BCL-2 antibody whereas milk blocking (5%) were used by CALU or Caspase 3 antibodies. Membranes were incubated with specific monoclonal caspase 3 (diluted 1:1000; #9661; Cell Signaling, Beverly, MA, USA), specific monoclonal BCL-2 (diluted 1:100; #sc-7382, SantaCruz), specific monoclonal Calumenin (diluted 1:30; sc- sc-271357) and monoclonal anti-α-tubuline antibodies (diluted 1:20000; Sigma-Aldrich, St. Louis, MO, USA), respectively, overnight at 4 °C with constant agitation. Following the incubation of the primary antibodies, membranes were washed and incubated for 2 h at room temperature with HRP-linked secondary anti-mouse IgG (diluted 1:10000; Cell Signaling, Beverly, MA, USA) or anti-rabbit IgG (diluted 1:5,000; Cell Signaling, Beverly, MA, USA), respectively. After washing, immunoreactive bands were visualized using Clarity Western ECL Substrate (Bio-Rad, Hercules, CA, USA). The immunoreactive bands were analyzed using a lab analysis software imaging densitometer (Bio-Rad, Hercules, CA, USA). α-tubuline and α-actin were used as loading and normalizing control.

### 2.16 Statistical analyses

Statistical analysis was performed using unpaired Student’s t-tests and ANOVA test as previously reported (García-Padilla et al., 2022; Garcia-Padilla et al., 2022a). Significance levels of p values are stated in each figure legend. p < 0.05 was considered statistically significant. Significance levels of SeaHorse analysis were performed using a ANOVA test.

## 3 Results

### 3.1 LncRNAs associated to Wnt signaling are deregulated in different models of cardiomyopathies

**Figure 1:**
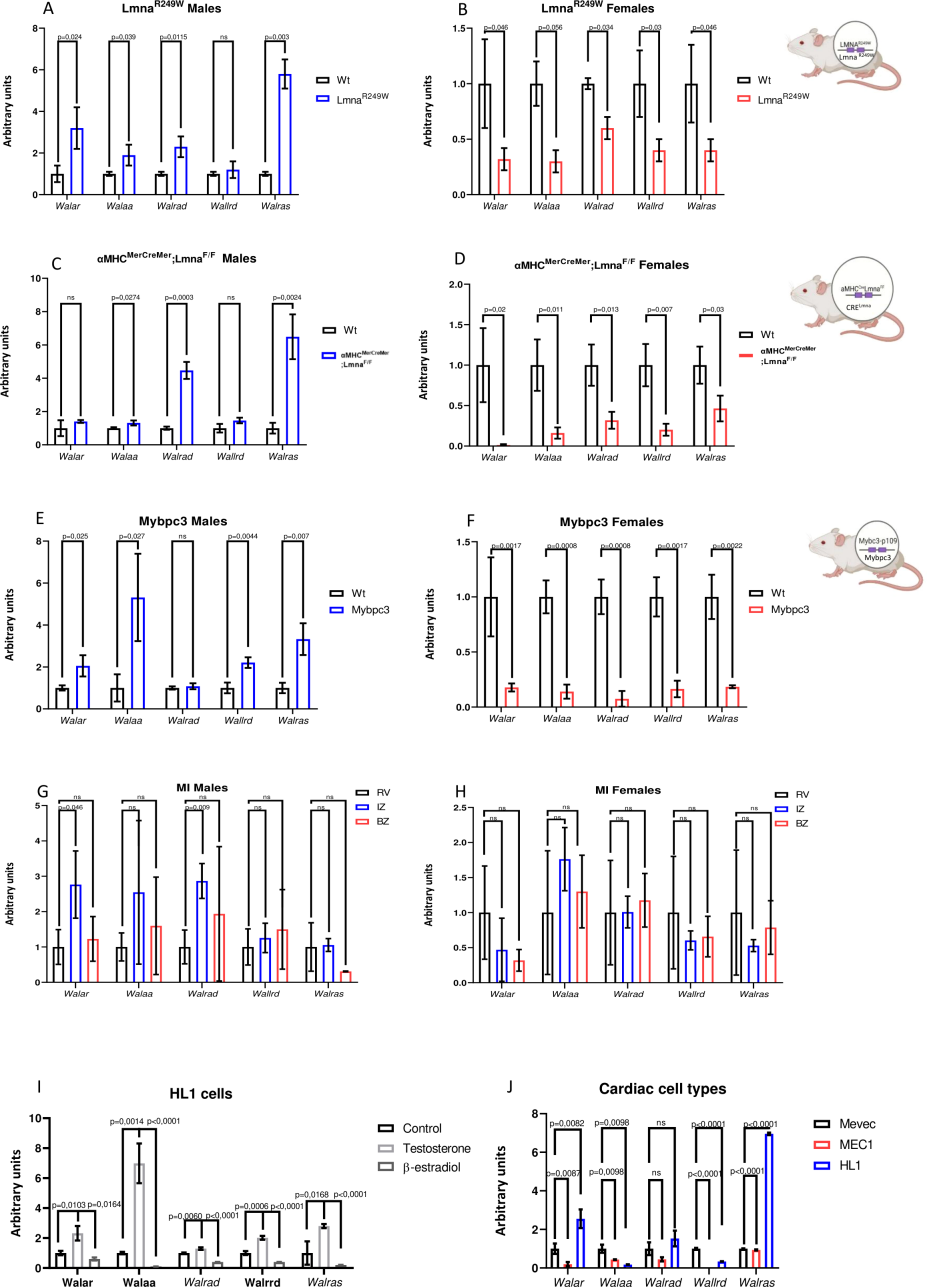
Walar, Walaa, Walrad, Wallrd and Walras expression analyses. RT-qPCR analyses of *Walar*, *Walaa*, *Walrad*, *Wallrd* and *Warlas* in LMNA^R249W^ **(A, B)** (n=3 per group), αMHC^MerCreMer^; LMNA^F/F^ **(C, D)** (n=3 per group) and Mybpc3 **(E, F)** (n=3 per group) left ventricles from male and female adult mice. RT-qPCR analyses of *Walar*, *Walaa*, *Walrad*, *Wallrd* and *Warlas* in dissected left ventricles –infarct zone (IZ), border zone (BZ) and remote zone (RZ)- from acute myocardial infarction **(G, H)** (n=3 per group). RT-qPCR analyses of *Walar*, *Walaa*, *Walrad*, *Wallrd* and *Walras* in β-estradiol treated HL1 cell line **(I)** (n=3 per group) and differential analyses expression of them in several cardiac cell types – cardiomyocytes cell line (HL1), endocardial cell line (MEVEC) and epicardial cell line (MEC)- **(J)** (n=3 per group). Note that expression of all of them is downregulated in LMNAR249W, αMHC^MerCreMer^; LMNA^F/F^ and Mybpc3 females mice. However, expression of *Walaa* and *Walras* is upregulated in all studied males’ mice. Observe that only *Walar* and *Walrad* display an upregulation of their expression in infarct zone compared to the remote zone of infarct from left ventricle. Note that β-estradiol treatment results in a reduced expression of all of them. Observe that *Walras* and *Walar* exhibit an enriched expression in cardiomyocyte cell line while *Walaa* and *Wallrd* display a preference endocardial expression. Finally, *Walrad* is expressed broadly in all cardiac cell types studied. Three biological samples were used in each qPCR analysis. Statistical analysis: Student’s t (95% confidence interval); p-value is described in each column.

Firstly, we explored the expression of this subset of lncRNAs associated to Wnt signaling – *Walar, Walaa, Walrad, Wallrd* and *Walras* – in dilated cardiomyophaty (DCM), dissecting their expression in two different DCM mouse models –*Lmna^R249W^* and *αMHC^MerCreMer^;Lmna^F/F^* –. Expression analysis *on Lmna^R249W^* mice demonstrated an upregulated expression of *Walar*, *Walaa*, *Walrad* and *Walras but not Wallrd* in males (**Figure 1A**) accompanied by downregulated expression of all of them in females (**Figure 1B**). Similarly, *αMHC^MerCreMer^;Lmna^F/F^* males displayed upregulation of *Walar*, *Walaa*, *Wallrd* and *Walras* (**Figure 1C**) while all of them are downregulated in *αMHC^MerCreMer^;Lmna^F/F^* females (**Figure 1D**). Interestingly, the opposed expression pattern expression detected in males and females suggests the existence of sex dependent regulation. Moreover, cardiac function analysis reveals that *αMHC^MerCreMer^; Lmna^F/^*female mice display a less severe cardiac dysfunction (**Supplementary Figure 1**).

To explore if the deregulated expression observed in males and females is specific for DCM mouse models or in contrast may be noticed in other cardiomyophaties, we examined the expression of these lncRNAs in a hypertrophic cardiomyopathy (HCM) mouse model -Mybpc3-p109-. The expression of *Walar*, *Walaa*, *Walrrd* and *Walras* is upregulated in *Mybcp3* males compared to control (wild type) mice (**Figure 1E**). Curiously, expression of all of them are downregulated in HCM females (**Figure 1F**) in line with the observation in DCM experimental models.

Furthermore, we analyzed if this subset of lncRNAs is modulated by an acute cardiac pathological process such as myocardial infarction (MI). Curiously, in this pathological context, only *Walar* and *Walrad* expression is upregulated in the infarct zone compared to remote zone of infarcted male hearts while the rest of the lncRNAs are not altered, neither in males nor in female infarcted hearts, suggesting that deregulation of lncRNAs analyzed is observed in chronic pathological processes such as cardiomyopathy but not in acute process such as myocardial infarction, for most of these lncRNAs (**Figures 1G, H**).

Since sex differences were observed, we tested if lcnRNAs expression would be regulated by female hormone β-estradiol and male hormone testosterone. *In vitro* analysis demonstrated that treatment of HL1 cell line with β-estradiol resulted in downregulation of all lncRNAs studied while testosterone treatment increased the expression of all of them supporting the regulation by sex hormones (**Figure 1I**).

Given the deregulated expression observed in dilated and hypertrophic cardiomyopathies of this subset of lncRNAs, we explored their expression in different cardiac cell types. qPCR analysis demonstrates that *Walras* and *Walar* displayed enriched myocardial expression, while in contrast, *Wallrd* and *Walaa* exhibited enriched endocardial expression and *Walrad* expression is detected broadly in epicardial, myocardial and endocardial cell types (**Figure 1J**).

### 3.2 *Walras* modulates apoptosis associated to UPR signaling

**Figure 2:**
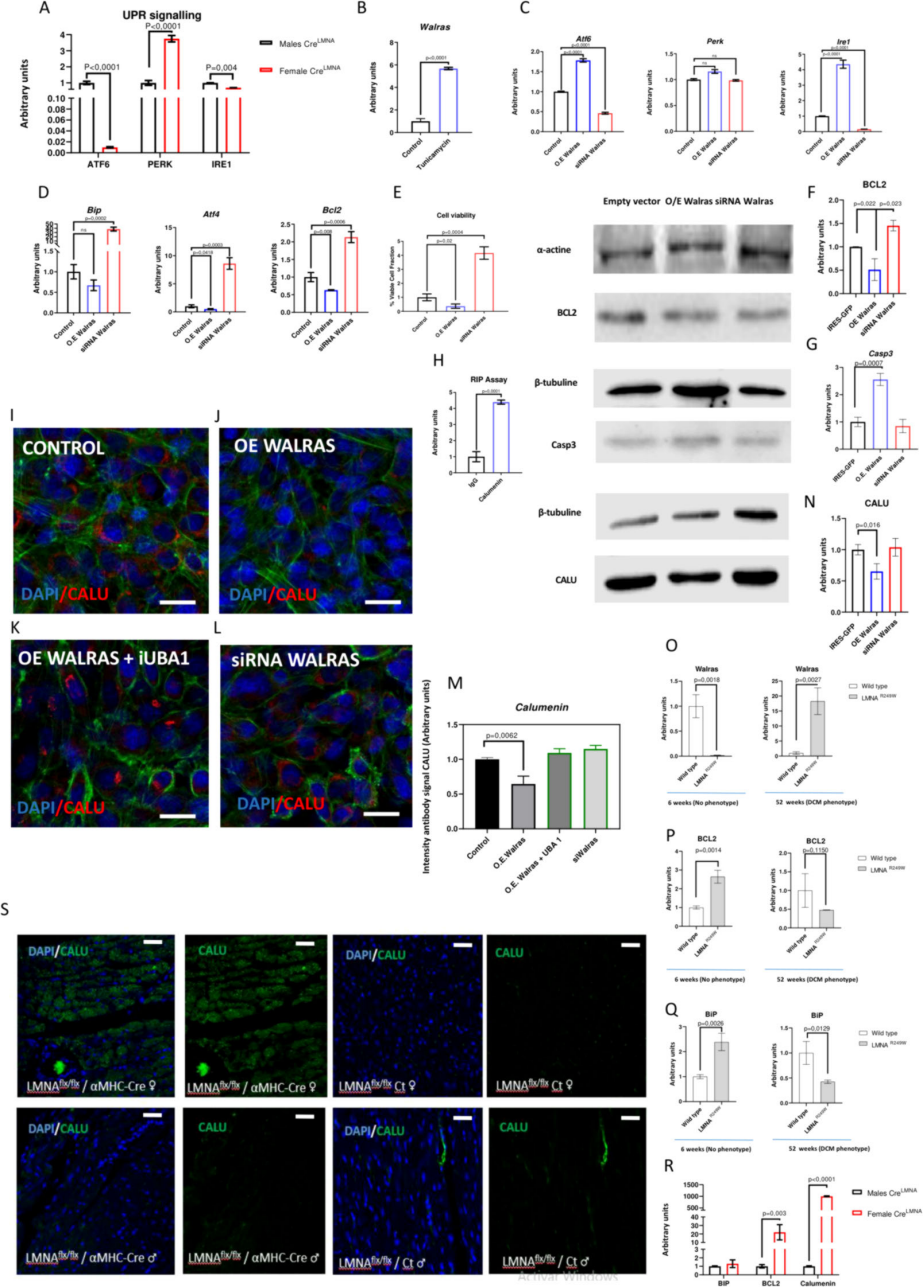
Analyses of UPR signalling in AMHCMerCreMer; LmnaF/F mices and his modulation by Walras functional assays. RT-qPCR analyses of *Atf6*, *Perk* and *Ire1* in Female and Males αMHCMerCreMer; LMNAF/F mices **(A)** (n=3 per group). Note that *Atf6* and *Ire1* are downregulated in females compared to males while expression of *Perk* is upregulated in females compared to males. RT-qPCR analyses of Walras in HL1 treated with tunicamycin **(B)** (n=3 per group). Observe that Walras expression is upregulated by triggering of UPR signalling. RT-qPCR analyses of *Atf6*, *Ire1*, *Perk*, *Bip*, *Atf4* and *Bcl2* gene expression in Walras gain or loss of function **(C, D)** (n=3 per group) Note that *Walras* modulates positively *Atf6* and *Ire1* whereas repress *Bip*, *Atf4* and *Blc2* expression. Cell viability assay after silencing or overexpression of Walras **(E)** (n=3 per group). Observe that Walras overexpression is translated into higher number of apoptotic cell whereas reduced number of apoptotic cells are observed in Walras siRNA treatment. Western Blot analyses of CASP3 and BCL2 protein levels in Walras gain or loss of function **(F, G)** (n=3 per group). Note that CASP3 protein levels is increased in Walras overexpression. Like CASP3 protein, BCL2 protein expression in reduced in Walras overexpression. RNA immunoprecipitation (RIP) of CALU following to RT-qPCR analyses **(H)** (n=3 per group). Observe that Walras is strongly enriched in the RNA fraction from CALU immunoprecipitation compared to IgG control. IMQ of CALU in *Walras* gain or loss of function and UBA1 treatment **(I–M)** (n=3 per group). Observe that CALU expression is reduced by *Warlas* overexpression and upregulated to siRNA Walras treatment while reduced expression observed in Walras overexpression is restored to control levels by UBA1 treatment. Furthermore, Western Blot of CALU demonstrates that expression of this protein is reduced in Walras overexpression **(N)** (n=3 per group). RT-qPCR analyses of *Walras*, *Bcl2 and Bip*, in in male LMNAR249W male heart samples before these mice developed the cardiac phenotype associated with this disease 6 weeks and subsequently 52 weeks Note that Walras in downregulated at in 6 weeks heart samples while it is upregulated at in 52 weeks. Unlike Walras, Bip and Bcl2 did notn’t show differences at in 6 weeks but they were downregulated in 52 weeks suggesting that Walras upregulation Walras is accompanied by of downregulation of antiapoptotic genes in DCM. RT-qPCR analyses of *Bip*, *Bcl2* and Calumenin in αMHCMerCreMer; LMNAF/F males and females mices **(O–R)** (n=3 per group). Observe that expression of three anti-apoptotic genes are downregulated in males compared to females. CALU expression in αMHCMerCreMer; LMNAF/F male and female and Wild type mouse. Note that CALU is upregulated in female compared to male αMHCMerCreMer; LMNAF/F and male and female Wild type mice **(S)** (n=3 per group). Three biological samples were used in each analysis. Statistical analysis: Student’s t (95% confidence interval p-value is described in each column. Scale Bars Heart tissue: 250 μM **(S)** Scale Bars HL1: 50 μm **(I–M)**.

Recently, a pivotal role of UPR signaling as a risk factor in the development of DCM has been reported (Al-Yacoub et al., 2021). To explore whether UPR signaling modulation underlies the sex differences observed in the occurrence of DCM in these experimental models, the expression of three pivotal genes – *Atf6, Ire1* and *Perk*- of UPR pathway activation were analyzed by RT-qPCR in *αMHC^MerCreMer^;Lmna^F/F^* male and female hearts, respectively. Curiously, *Atf6* and *Ire1* expression is downregulated in *αMHC^MerCreMer^;Lmna^F/F^* females as compared to *αMHC^MerCreMer^;Lmna^F/F^* males whereas that *Perk* expression is upregulated in *αMHC^MerCreMer^;Lmna^F/F^* females compared to C *αMHC^MerCreMer^;Lmna^F/F^* males (**Figure 2A**), supporting thus sex differences in the UPR pathway in this DCM experimental model. Since *Walras* displays an enriched myocardial expression and is downregulated in DCM females but upregulated in DCM males, we focused our efforts on dissecting a possible participation of this lncRNA in the UPR signaling and therefore ERS process. Firstly, we checked if *Walras* expression would be modulated by ERS activation. HL1 treated with tunicamycin, a known UPR activator, revealed a marked upregulation of *Walras* suggesting a possible role in the UPR signaling (**Figure 2B**). Hence, gain and loss of function essays were performed to determinate the role of *Walras* in the triggering of UPR pathway. Curiously, *Walras* gain of function resulted in upregulation of *Atf6* and *Ire1* whereas that *Walras* inhibition is translated into downregulation of these two factors (**Figure 2C**). Furthermore, *Walras* silencing resulted in upregulation of *Bip*, *Atf4* and *Bcl2* whereas *Walras* gain of function resulted in downregulation of these three anti-apoptosis genes (**Figure 2D**). Consequently, analysis of cellular viability by flow cytometry demonstrated an increased number of viable cells in *Walras* loss of function compared to controls. In contrast, *Walras* gain of function was translated into a decreased of viable cells and increased number of death cells (**Figure 2E****).** In line with these findings, Western blot analysis demonstrated that gain of function of *Walras* increased caspase 3 protein level while decrease the Bcl2 protein levels (**Figures 2F, G**). Therefore, our data demonstrate that *Walras* modulates UPR pathway, followed an increased apoptosis in HL1 cell line, suggesting thus an enhanced pro-apoptotic UPR signaling pathway.

Previously, we have described that *Walras* may physically interact with calumenin (CALU) protein, an anti-apoptotic factor in UPR context (García-Padilla et al., 2022). Herein we demonstrated by RNA immunoprecipitation (RIP) such physical interaction between *Walras* and CALU (**Figure 2H**). Furthermore, immunohistochemical (IMQ) analysis revealed that *Walras* silencing and overexpression resulted in increased and reduced CALU protein levels, respectively, as compared to controls (**Figures 2I–L**), suggesting that the interaction between *Walras* and CALU promotes the degradation of this protein and consequently, reduces the cellular viability. To determine if observed CALU degradation by *Walras* could be mediated by ubiquitination, we blocked the function of Ubiquitin Like Modifier Activating Enzyme (UBA1), a pivotal E3 ligase (García-Padilla et al., 2022). *Walras* gain of function in HL1, followed by UBA1/E1 enzymatic inhibition resulted in absence of CALU protein levels, compared to controls, suggesting that CALU degradation by *Walras* is mediated by ubiquitination process (**Figures 2I–N**).

Curiously, *Walras* gain of function in primary mouse E18.5 and neonatal cardiomyocyte cultures increased the expression of *Atf6*, *Ire1* and *Perk*, similarly as *Walras* overexpression in HL1 cells. Furthermore, the expression of *Bip* and *Atf4* is upregulated in both cardiomyocyte cultures (ED.18,5 and neonatal stages) whereas *p53*, *Chop* and *Xbp1* expression is downregulated in neonatal cardiomyocytes but upregulated in ED18.5 cultures. In contrast to *Walras* overexpression in HL1, gain of function of *Walras* in both primary cardiomyocyte cultures the expression of *Bcl2* and *Calu* is increased, suggesting that *Walras* may modulate a protective role against UPR signaling in this context. To determinate if protective effect of *Walras* overexpression is characterize of primary cardiomyocyte or by the contrary is since a fibroblast presence in the cardiomyocyte culture, we isolated primary cardiac fibroblast and carried out *Walras* gain of function essays demonstrating that the *Walras* overexpression in cardiac fibroblast results in an increase expression of Bcl2 and Calu suggesting that possible protective role of Walras in cardiomyocyte culture may be partially explain by the cardiac fibroblast (**Supplementary Figure 2**).

Finally, to explore if *Walras* expression and antiapoptotic pathway is activated before the appearance of DCM phenotype, we analyzed the expression of *Walras*, *Bip* and *Bcl2* in *LMNA^R249W^* male heart samples before these mice developed the cardiac phenotype associated with this disease -6 weeks- and subsequently -52 weeks-. qPCR analysis demonstrated that *Walras* in downregulated at 6 weeks heart samples while it is upregulated at 52 weeks. Unlike *Walras*, *Bip* and *Bcl2* did not show differences at 6 weeks but they were downregulated at 52 weeks suggesting that *Walras* upregulation is accompanied by downregulation of antiapoptotic genes in DCM (**Figures 2O–Q**). Furthermore, we determined the expression of *Calu* and *Blc2* RNA levels in DCM males and females demonstrating that expression of both anti-apoptotic genes is upregulated in females compared to males. These data suggest that apoptosis may be reduced in DCM females resulting therefore in more protected cardiac function (**Figure 2R**). Since, the physical interaction described between *Walras*-CALU is at protein level, we explore the expression of this protein in female *αMHC^MerCreMer^;Lmna^F/F^* and male *αMHC^MerCreMer^;Lmna^F/F^* heart samples. Our results show that CALU is highly detected in *αMHC^MerCreMer^;Lmna^F/F^* females but neither in *αMHC^MerCreMer^;Lmna^F/F^* males nor in control female and male hearts samples (**Figure 2S**). These data together with the downregulation of *Walras* expression in *αMHC^MerCreMer^;Lmna^F/F^* females but not in males, supports the notion of a functional role of *Walras* in repression of this anti-apoptotic protein, pinpointing thus as a deleterious factor in DCM.

Correct expression of desmosomal proteins are essential to proper cell-to-cell adhesion within the cardiomyocytes (Garcia-Pavia et al., 2011; Smith et al., 2020). Alterations in several desmosomal proteins such as desmoplakin or plakoglobin are translated into arrhythmogenic dysfunctions and dilated cardiomyopathies. Furthermore, different mutations in desmoplakin are linked to dilated cardiomyopathy demonstrating that desmosomal instability is a cardiomyocyte feature in DCM (Rampazzo et al., 2002; Elliott et al., 2010; Gómez-Domínguez et al., 2020; Hubert et al., 2022; Karvonen et al., 2022). We therefore explored if *Walras* can modulate the desmoplakin and plakoglobin signaling. No change in the pivotal genes involved in these pathways are observed by gain or loss of function of *Walras* suggesting that cell-cell adhesion is not modulated by *Walras* (**Supplementary Figure 3**).

### 3.3 *Walras* alters mitochondrial function by modulating mitochondrial mass

**Figure 3:**
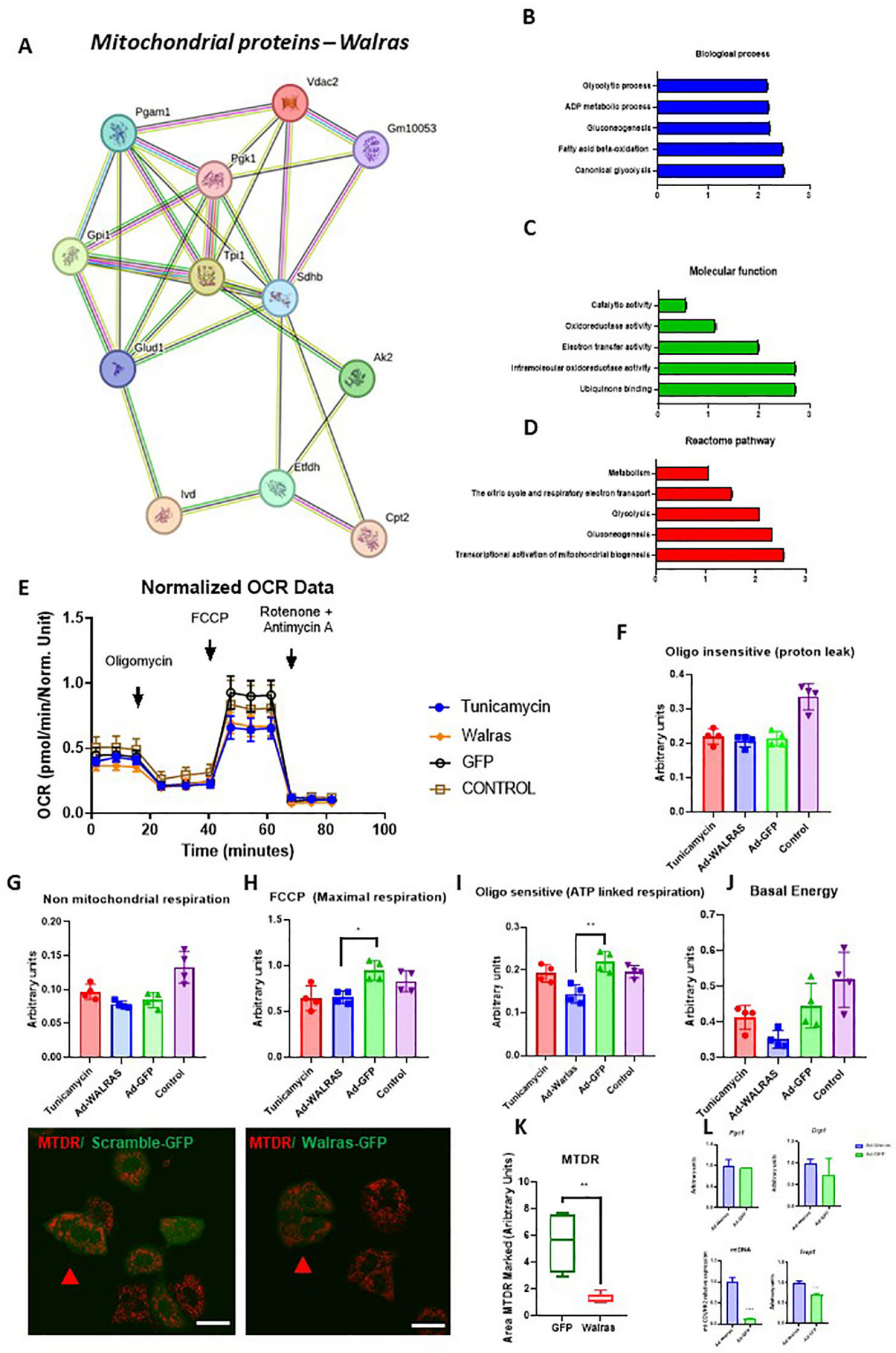
Analysis of mitochondrial activity modulation by Walras functional assay. Protein-protein interaction (PPI) of mitochondrial protein associated to Walras **(A–D)** (n=3 per group); Note that Walras interact with several mitochondrial proteins involved in the mitochondrial respiration such as electron transfer activity or intramolecular oxidoreductase activity. OCR analyses in Walras overexpression and Tunicamycin treatment **(E–I)** (4 technical replicates by observed parameter). ANOVA analysis was used to stadistic significance of OCR essay. Observe that maximal respiration and ATP linked respiration is reduced in Walras overexpression whereas tunicamycin treatment reduces maximal respiration but not affect to ATP linked respiration. IMQ of MTDR protein in Walras overexpression **(K)** (4 technical replicates by observed parameter). Note that cardiomyocytes infected with Ad-Walras-GFP (green cells) display a lower expression to MTDR (red signals) compared to cell infected with Ad-Scramble-GFP. RT-qPCR analyses of Pgc1, Drp1, Trap1 and mtDNA **(L)** (n=3 per group). Note to Trap1 expression is upregulated in Walras overexpression while Pgc1 and Drp1 expression not display changes. Furthermore, mtDNA amount is strongly increased in Walras overexpression. Three biological samples were used in each analysis. Statistical analysis: Student’s t and ANOVA analysis; (95% confidence interval); p-value is described in each column. Scale Bars HL1 cell line: 25 μM **(K)**. * is p<0,05 and ** is p<0,01.

A proper crosstalk between mitochondria and RE is pivotal to maintain cellular homeostasis (Veeresh et al., 2019). Disruption of mitochondrial activity results in an increased cellular stress triggering UPR signaling in cardiomyocytes (Ramaccini et al., 2021). Furthermore, several mitochondrial alterations have reported in the pathogenesis of DCM (Shpilka and Haynes, 2018). Previously, we demonstrated that *Walras* may interact with 12 mitochondrial proteins including Voltage Dependent Anion Channel 2 (VDAC2), Phosphoglycerate Mutase 1 (PGAM1), Phosphoglycerate kinase 1 (PGK1), Glucose-6-Phosphate Isomerase (GPI1), triosephosphate isomerase 1 (TPI1), Succinate Dehydrogenase Complex Iron Sulfur Subunit B (SDHB), Glutamate Dehydrogenase 1 (GLUD1), Adenylate Kinase 2 (AK2), carnitine palmitoyltransferase II enzyme (CPT2), Electron-Transferring-Flavoprotein Dehydrogenase (ETFDH) and Isovaleryl-CoA Dehydrogenase (IVD) (García-Padilla et al., 2022). *Walras* associated proteins preferentially participate in ubiquinone binding and in several mitochondrial activities such as oxidoreductase, electron chain transport and catalytic functions. Furthermore, these proteins are involved in several energetic processes such as glycolysis, fatty acid beta-oxidation and gluconeogenesis (**Figures 3A–D**). To elucidate if *Walras* may modulate mitochondrial function, *Walras* gain of function in HL1 cell line followed by SeaHorse analysis was performed. *Walras* overexpression resulted in a decrease of maximal respiration and ATP linked respiration suggesting that *Walras* may negatively affect mitochondrial respiration. In addition, tunamycin treatment decreased maximal respiration but did not affect ATP linked respiration, suggesting that *Walras* may modulate maximal mitochondrial respiration by inducing ERS (**Figures 3E–J**). Consequently, *Walras* upregulation drastically reduces mitochondrial area compared to controls, suggesting that reduction of maximal and ATP linked respiration may be caused by loss of mitochondrial area (**Figure 3K**). Furthermore, we explore if lower mitochondrial area associated with *Walras* overexpression was caused by alteration in mitochondrial biogenesis and/or fission. qPCR analysis demonstrates that expression levels of *Pgcα1* and *Drp1* do not show changes. However, mitochondrial DNA is increased in *Walras* gain of function. Finally, we tested the expression of *Trap1*, a known mitochondrial chaperone, demonstrating upregulated expression after *Walras* overexpression (**Figure 3L**). Therefore, these data suggest that *Walras* gain of function may increase mitochondrial stress accompanied by lower respiration capacity of mitochondria. Furthermore, Walras overexpression increases Trap1 expression suggesting a stress mitochondrial condition in response to Walras. On the other hand, the increased amount of mtDNA should be address in future studies.

### 3.4 *Walras* human homologues modulate UPR signaling

**Figure 4:**
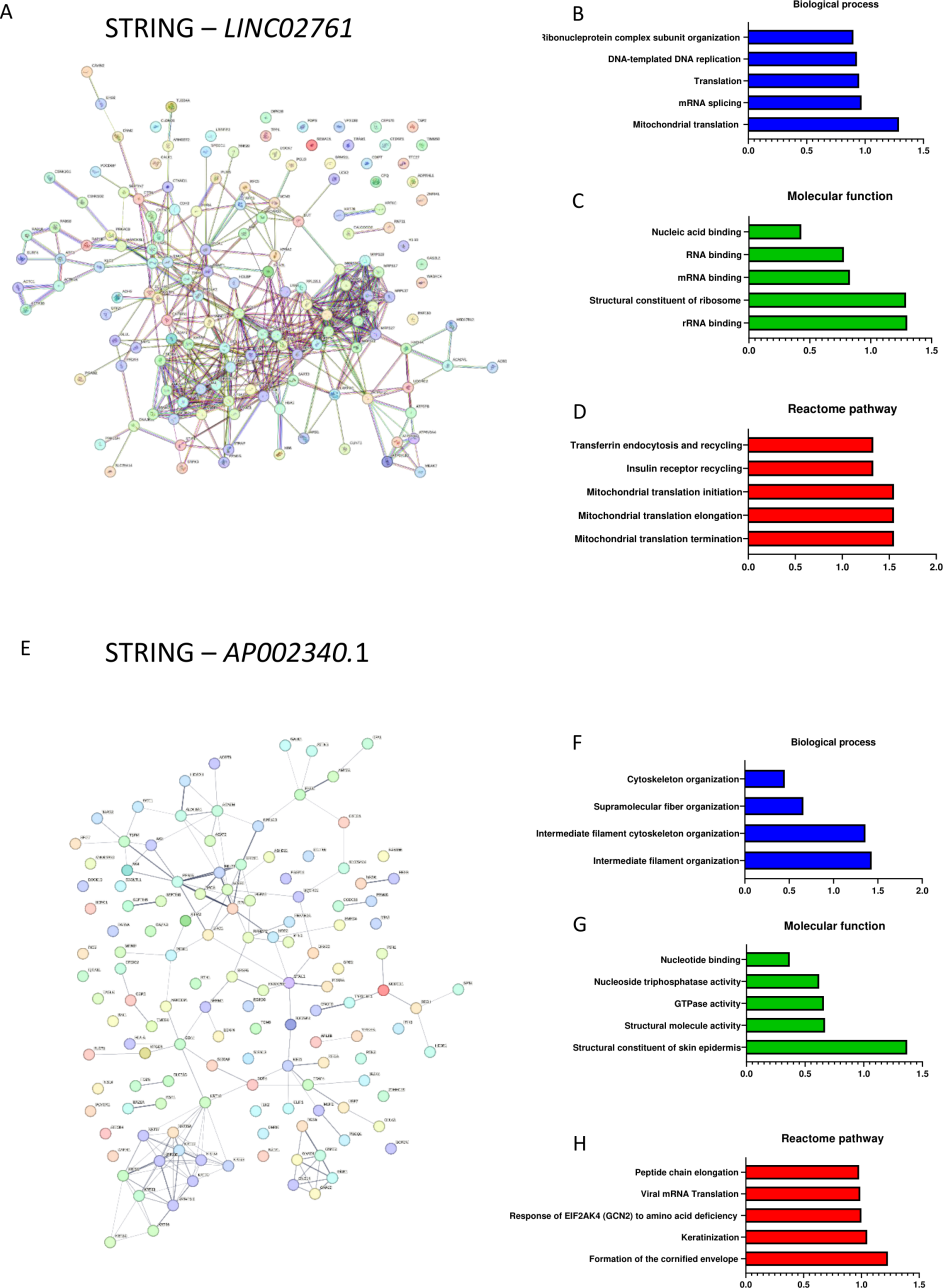
Interaction and functional enrichment analyses of proteome associated to LINC02761 and AP002340.1. Protein-protein interaction (PPI) of proteome associated to LINC02761 **(A–D)**; Protein-protein interaction (PPI) of proteome associated to AP002340.1 **(E–H)** (n=3 per group). Note to LINC02761 interact with several mitochondrial proteins involved in translation process while AP002340.1 is related to cytoskeleton proteins associated to intermediate filament organization.

We have previously described two potential *Walras* human homologues lncRNAs, *LINC02761* and *APO02340.1* (García-Padilla et al., 2022). To identify putative common pathways between *Walras* and *LINC02761* and/or *APO02340.1*, biotinylated RNA probes were generated, pulldown assays followed by MS were performed. Proteome analysis demonstrates that *LINC02761* interacts with 147 proteins. *LINC02761* associated proteins are involved in several biological processes such as mitochondrial translation, mRNA splicing and translation, DNA-templated DNA replication and ribonucleoprotein complex subunit organization (**Figures 4B–D**). Furthermore, reactome pathway analysis proved that *LINC02761* interacts with several proteins required for proper mitochondrial translation elongation, initiation and termination in line with its cytoplasmatic expression (**Figure 4A**). Proteome analysis of *APO02340.1* demonstrates interaction with 107 proteins involved in cytoskeleton organization, supramolecular fiber organization, intermediate filament cytoskeleton organization and intermediate filament organization (**Figures 4F–H**). Furthermore, reactome pathway analysis reveals to *APO02341.1* interacts with different proteins required to peptide chain elongation and formation of the cornified envelope (**Figure 4E**). Comparative analysis of interacting proteome associated to *Walras*, *LINC02761* and *APO02340.1* demonstrates that no protein interaction is shared between them although they have a high degree of similarity in their nucleotide sequence (**Supplementary Figure 4**). Complete list of interacting proteins from each lncRNA - *LINC02761* or *APO02341.1* is described in **Supplementary Tables 2**, **3** respectively.

**Figure 5:**
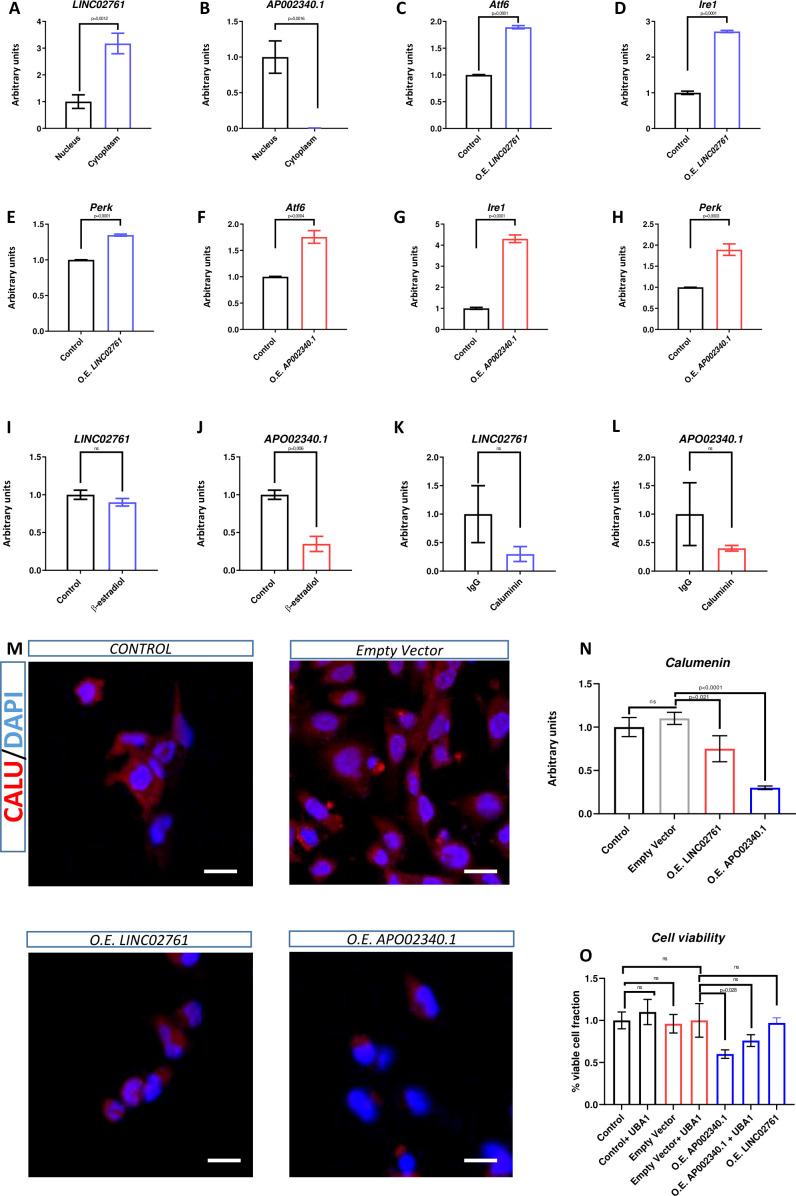
Molecular characterization of LINC02761 and APO02340.1. RT-qPCR analysis o LINC02761 and APO02340.1 in AC16 cytoplasmatic and nuclear fraction **(A, B)** (n=3 per group). Observe that LINC02761 expression is enriched in cytoplasmatic fraction whereas APO02340.1 expression is enriched in nuclear fraction of AC16. RT-qPCR analysis of Atf6, Ire1 and Perk in LINC02761 and APO02340,1 gain of function **(C–H)** (n=3 per group). Note that LINC02761 and APO02340,1 overexpression are translated into upregulation of Atf6, Ire1 and Perk. RT-qPCR analysis of LINC02761 and APO02340.1 expression in AC16 treated with β.estradiol **(I, J)** (n=3 per group). Observe that APO02340,1 expression is downregulated compared to control but not LINC02761 expression. RIP assay followed to RT-qPCR analyses of LINC02761 and APO02340.1 **(K, L)** (n=3 per group). Observe that both lncRNAs not interact directly with CALU protein. IMQ against CALU **(M, N)** (n=3 per group). Note that protein levels of CALU are reduced by LINC02761 and APO02340,1 overexpression. Cell viabilitiy assay followed to flox cytometry **(O)** (n=3 per group). Observe that APO02340.1 overexpression results in increased number of apoptotic cells. Furthermore, treatment with UBA1 reduces apoptosis cellular observed in APO02340.1 overexpression. Three biological samples were used in each analysis. Statistical analysis: Student’s t (95% confidence interval); p-value is described in each column. Scale Bars AC16 cell line: 50 μM **(M)**.

Even though that *Walras*, *LINC02761* and *APO02340.1* do not share the same interacting proteins, we investigated whether they could exert the same or similar function. Firstly, we checked the subcellular expression of both lncRNAs in human cardiomyoblast cell line (AC16). qPCR analysis expression in AC16 revealed that *LINC02761* displays a cytoplasm enriched expression whereas *AP002340.1* locates to the nucleus, similarly to *Walras* expression (**Figures 5A, B**). *LINC02761* gain of function in AC16 cells resulted in increased *Atf6* and *Ire1* mRNA expression while no difference was observed for *Perk*. On the other hand, *APO02340.1* gain of function in AC16 cells resulted in increased levels of *Atf6*, *Ire1* and *Perk* mRNA expression suggesting that both lncRNAs may be modulating the activation of the UPR pathway in AC16 cells (**Figures 5C–H**). To address if expression of *LINC02761* and *APO02340.1* is dependent of sex hormones, AC16 cells were treated with β-estradiol, demonstrating that *AP02340.1*, but not *LINC02761*, is downregulated, in line with the observations in HL1 cells after *Walras* overexpression (**Figures 5I, J**).

To analyze if *LINC02761* or *APO02340.1* may modulate apoptosis associated to UPR signaling, we assessed the possible interaction between calumenin protein and both lncRNAs. RIP assay demonstrates that calumenin protein does not interact directly with any of these lncRNAs (**Figures 5K, L**). However, gain of function of *LINC02761* or *APO02340.1* resulted in decreased levels of calumenin protein suggesting that both lncRNAs indirectly modulate protein expression of this anti-apoptotic factor (**Figure 5M**). In line with these results, the number of viable cells is reduced in *AP002340.1*, but not in *LINC02761* overexpression. However, unlike *Walras*, the number of viable cells does not increase when UBA1 is inhibited, suggesting the existence of a more complex mechanism than that mediated by *Walras* in mice (**Figures 5N, O**). These data suggest that although both lncRNAs indirectly modulate calumenin protein levels, only *APO02340.1*-calumenin indirected interaction exert an effect that promotes cellular apoptosis.

## 4 Discussion

Several studies have demonstrated the pivotal role of distinct lncRNAs in cell homeostasis and their contribution to the development of distinct cardiovascular disorders (CVDs) (Garcia-Padilla et al., 2022b; Kohlmaier et al., 2023; Martinez-Amaro et al., 2023; Yaghoobi et al., 2024). Dilated Cardiomyopathy (DCM) is a disease of the heart muscle characterized by enlargement and dilation of one or both ventricles along with impaired contractility defined as left ventricular ejection fraction (LVEF) less than 40% (Schultheiss et al., 2019). Recently, different studies have pinpointed the contribution of the reticulum endoplasmatic stress (ERS) in the pathogenesis of this disease (Castillero et al., 2015; Al-Yacoub et al., 2021; Qiu et al., 2021). However, the underlying mechanisms leading to DCM remain poorly understood, requiring therefore further analysis. In our study we demonstrated a sex dependent expression of a subset of lncRNAs- *Walar*, *Walaa*, *Walrad*, *Wallrd* and *Walras*-, previously described in the context of AF, in several models of DCM and HCM. Furthermore, such a sex dependent modulation is not specific of the type of cardiomyopathy and is regulated by a β-estradiol and testosterone hormones. In this context, it is important to highlight that several studies reported higher prevalence of cardiac disorders in men compared to women (McNamara et al., 2011; Stanhewicz et al., 2018; Bajelan et al., 2019; De Bellis et al., 2020; Roth et al., 2020; Walker et al., 2021). Additionally, men display more severe clinical manifestations accompanied by a worse outcome and improvement of several cardiac diseases that women (van Rijsingen et al., 2013; Doesch et al., 2014; Li et al., 2015; Halliday et al., 2018; Thapa et al., 2019). Interestingly, such sex differences equalize once women reach menopause and their hormonal levels decrease, supporting a protective role of female hormones against cardiac disoders (Iorga et al., 2017; Newson, 2018; Mathews et al., 2019; Zhu et al., 2019; Ryczkowska et al., 2022). On the other hand, expression analysis of these lncRNAs in acute myocardial disease displayed no significant differences, except for *Walar* and *Wallrd*, suggesting that deregulation is not present in such acute disease model. DCM and HCM are chronic pathologies while myocardial infarction is an acute pathological process. Molecular mechanisms governing both processes are different and the implications of the same signaling pathways in both processes may be opposite. For example, ERS and UPR signaling negatively affect various CVDs such as atherosclerosis, cardiac hypertrophy and dilated cardiomyopathy, and such chronic stress is associated to increased myocardial apoptosis (Minamino et al., 2010; Hong et al., 2017). However, recent evidences have proved that ERS activation in acute myocardial infarction has beneficial effect, inducing better damaged tissue repair (Wu et al., 2024). This opposite role of ERS and UPR signaling in apoptosis induction may explain the dysregulation observed of *Walras* in chronic diseases – DCM and HCM- but not in acute myocardial infarction.

The main cell type affected in DCM is the cardiomyocyte, which frequently displays an irregular hypertrophy and higher rate of apoptosis (Ito et al., 2021). Furthermore, different studies have demonstrated that ERS mechanism and UPR signaling govern cardiomyocyte apoptosis associated to DCM and exert a harmful role in this process pinpointing to cardiomyocyte as the pivotal cardiac cell type involved in this pathological process (Al-Yacoub et al., 2021; Toro et al., 2022). Given the high expression of *Walras* in HL1 cardiomyocytes and its deregulation both DCM models we focused our attention in this lncRNA as further candidate to explore its role in DCM.

Our results evidence that *Walras* activates UPR signaling by promoting *Atf6* and *Ire1* expression. Both factors are related with increased expression of pro-apoptotic factors and repression of protective genes in cardiomyocytes (Toro et al., 2022). As results of UPR activation by *Walras*, higher number of apoptotic cardiomyocytes were observed, suggesting that it may promote the apoptosis associated to ERS and thus exerting a negative role in cardiomyocyte cellular homeostasis. Previously, we described by MS a subset of HL1 proteins that interact with *Walras* in cardiomyocytes, among which are different proteins associated with cellular apoptosis such as CALU or CATD (Lenin et al., 2019; García-Padilla et al., 2022; Chen et al., 2023). Mechanistically, we demonstrated that *Walras* physically interacts with CALU, reducing its protein levels by UBA1 associated degredation (Shu et al., 2018). Several authors have described a protective role of CALU protein in apoptosis induced by UPR triggering (Lee et al., 2013; Wang et al., 2017). Curiously, the effect of *Walras* overexpression in primary cultures of cardiomyocytes from embryonic and neonatal stages differs from that observed in HL1 cell line, where it exerts to protective role by activating pro-survival signals associated to UPR pathway. These divergent effects may be due in respond to *Walras* overexpression in the remnant fibroblasts of the primary culture, which exert a positive effect against apoptosis.

Defects in mitochondrial function are associated with DCM and HCM pathogenesis (Senft and Ronai, 2015; Shpilka and Haynes, 2018; Zhang et al., 2019). MS analysis demonstrated that *Walras* may interact with several mitochondrial proteins. Reduced mitochondrial respiration activity accompanied to lower mitochondrial amount in *Walras* overexpression assays proved that this lncRNA is required to correct mitochondrial homeostasis. Several studies have proved that mitochondrial activity is reduced in cardiomyocyte harboring different mutations -i.e T12297C mutation in the mtDNA-tRNALeu (CUN) or mutations involving the evolutionarily conserved residues of cytochrome c oxidase subunit I, NADH dehydrogenase 5, tRNA^Ala^ and tRNA^Arg^- associated to DCM pinpointing the mitochondrial alteration in the pathogenesis of this disorder (Li et al., 1997; Grasso et al., 2001; Viola et al., 2013; Viola et al., 2014; Li et al., 2019; Ramaccini et al., 2021; Wang et al., 2022). Furthermore, upregulation of *Trap1* by *Walras* gain of function is consistent with an increased UPR signalling (Takemoto et al., 2011; Baqri et al., 2014; Xie et al., 2021)thus reinforcing the role of *Walras* in increasing the stress of the endoplasmic reticulum.

Previously, we have described that *Walras* have two human homologues lncRNAs – *APO02340.1* and *LINC02761*-. Common interacting proteins and/or the participation in the same biological process were analyzed between three lncRNAs after pulldown assays and mass spectrometry analyses, respectively. Nevertheless, our results demonstrated that both lncRNAs activate the UPR signaling while only *APO02340.1* increased the number of apoptotic cardiomyoblasts by reducing the expression of CALU protein, in a similar manner as observed for *Walras*. *APO02340.1*, interacts with different proteins involved in cytoskeleton organization and different mutations in these cytoskeleton proteins are translated into loss of cellular integrity of several cellular types and increasing ERS (Okada et al., 2004; Wilson and González-Billault, 2015; van Vliet et al., 2017; Gruber et al., 2023; Folahan et al., 2024). On the other hand, *LINC02761* interacts with mitochondrial proteins. Several authors have linked the correct mitochondrial homeostasis with proper ERS, demonstrating that alteration in mitochondrial proteins results in an increased ERS and consequently activation of UPR signalling (Viola et al., 2013; Viola et al., 2014; Senft and Ronai, 2015; Li et al., 2019; Shi et al., 2025). Our data suggest that both human *Walras* homologue lncRNAs may modulate UPR signaling by activation or repression of different molecular pathways as compared to *Walras.* In line with this notion, Chen et al. (2016) postulate that lncRNAs conserved across species may have a similar function by exerted by different pathways (Chen et al., 2016), in line with our findings.

Taken all together, we have demonstrated that a subset of lncRNAs previously described in an AF context, are also deregulated in DCM and HCM but not in acute myocardial infarction. Furthermore, expression of these lncRNAs is repressed by β-estradiol. Importantly, *Walras*, displays enriched expression in cardiomyocytes and *Walras* repression can trigger UPR signaling and apoptosis by modulating CALU anti-apoptotic protein levels throughout the proteasome pathway. In addition, we have evidenced that *Walras* alters mitochondrial activity and mass, contributing to the loss of cardiomyocyte homeostasis and increased to UPR response. Finally, we have demonstrated that *APO02340.1* a human Walras homologue lncRNA exerts a similar function in AC16 cell line suggesting a conservation functional role of *Walras* across mouse and human species.

Our results open the possibility of using Walras as a potential therapeutic target by blocking the function of estrogens and testosterone in dilated cardiomyopathy or other cardiomyopathies as promising solution for modulating the hormone-dependent deleterious effects in these diseases. Our data demonstrate that Walras and its human homologs could be administered in a clinical setting to attempt to positively modulate the anti-apoptotic pathway dependent on UPR signaling activation in the first 24 hours post-cardiac injury, in an attempt to protect cardiomyocytes from cell death associated with this injury. However, further studies are required to confirm and evaluate this therapeutic use.

## Data Availability

The datasets presented in this study can be found in online repositories. The names of the repository/repositories and accession number(s) can be found in the article/**Supplementary Material**.

## Supplementary material

Supplementary Data 1 Echography heart analysis of αMHCCre LmnaFF + TAM miles and mices. Left ventricular systolic volumen and Ejection Fraction of αMHCCre LmnaFF + TAM mices at day 0 and 4 after Tamoxyfen administration (Panels A-B) (n=3 per group). Note that LVVs is increased in males compared to females whereas ejection fraction is lower in males compared to females. Three biological samples were used in each analysis. Statistical analysis: Student’s t (95% confidence interval); * p-value < 0.05; ** p-value < 0.01; *** p-value < 0.001; **** p-value < 0.0001.

Supplementary Data 2 RT-qPCR analysis of Walras’ expression in gain and loss of function. Note that Walras expression is upregulated in gain of function analysis where expression of it is reduced in the loss of function analysis. Three biological samples were used in each analysis. Statistical analysis: Student’s t (95% confidence interval); * p-value < 0.05; ** p-value < 0.01; *** p-value < 0.001; **** p-value < 0.0001.

Supplementary Data 3 RT-qPCR analysis of Walras gain of function in primary cardiomyocytes and cardiac fibroblast over UPR signaling genes. Note that Walras gain of function in primary cardiomyocytes increased the expression of of *Atf6*, *Ire1* and *Perk*. Also, *Bip* and *Atf4* is upregulated in both cardiomyocyte cultures (ED.18,5 and neonatal stages) whereas *p53*, *Chop* and *Xbp1* expression is downregulated in neonatal cardiomyocytes but upregulated in ED18.5 cultures. Furthermore, the expression of *Bcl2* and *Calu* is increased, By other hand, *Walras* overexpression in cardiac fibroblast results in an increase expression of Bcl2 and Calu suggesting that possible protective role of Walras in cardiomyocyte culture may be partially explain by the cardiac fibroblast. Three biological samples were used in each analysis. Statistical analysis: Student’s t (95% confidence interval); * p-value < 0.05; ** p-value < 0.01; *** p-value < 0.001; **** p-value < 0.0001.

Supplementary Data 4 RT-qPCR analysis of desmoplakin and plakoglobin signalling. Note to expression of all of them is not alterated by Walras overerexpression (n=3 per group). Three biological samples were used in each qPCR analysis. Statistical analysis: Student’s t (95% confidence interval); * p-value < 0.05; ** p-value < 0.01; *** p-value < 0.001; **** p-value < 0.0001.

Supplementary Figure 5 Venny’s diagrams of LINC02761, Walras and APO02340.1. Observe that any protein is shared by LINC02761, APO02340.1 and Walras (n=3 per group).
