# Correction: Walras modulates sex-dependent endoplasmic reticulum stress in cardiomyopathy

**DOI:** 10.3389/fphys.2026.1904886

**Published:** 2026-07-06

**Authors:** Francisco J. Martinez-Amaro, Carlos Garcia-Padilla, Fernando Bonet, Elena Alonso-Villa, Rocio Toro, Houria Daimi, Eva Vargas, Ivan Hernandez, Borja Vilaplana-Martí, Ignacio Perez de Castro, Laetitia Bouchard, Fabien Hubert, Francesca Rochais, Antoine Muchir, Michaela Veliova, José Antonio Enriquez, Alejandro Salguero, Jose Luis De La Pompa, Estefania Lozano-Velasco, Diego Franco

**Affiliations:** 1 Cardiovascular Research Group, Department of Experimental Biology, University of Jaen, Jaen, Spain; 2 Medina Foundation, Technology Park of Health Sciences, Granada, Spain; 3 Department of Medicine, School of Medicine, University of Cadiz (UCA), Cadiz, Spain; 4 Research Unit, Biomedical Research and Innovation Institute of Cadiz (INiBICA), Puerta del Mar University Hospital, Cadiz, Spain; 5 Institute of Applied Molecular Medicine (IMMA), Department of Basic Medical Sciences, Facultad de Medicina, Universidad San Pablo-CEU, CEU Universities, Madrid, Spain; 6 Laboratory of Human Genome and Multifactorial Diseases (LR12ES07), Faculty of Pharmacy, University of Monastir, Monastir, Tunisia; 7 Systems Biology Unit, Department of Experimental Biology, University of Jaen, Jaen, Spain; 8 Instituto de Salud Carlos III, Madrid, Spain; 9 Aix-Marseille Univ, MMG, Inserm U1251, Marseille, France; 10 Sorbonne Université, INSERM U974, Institute of Myology, Center of Research in Myology, Paris, France; 11 Centro Nacional de Investigaciones Cardiovasculares Carlos III, Madrid, Spain; 12 CIBER de Fragilidad y Envejecimiento Saludable (CIBERFES), Madrid, Spain; 13 Intercellular Signaling in Cardiovascular Development and Disease Laboratory, Centro Nacional de Investigaciones Cardiovasculares Carlos III, Madrid, Spain; 14 Ciber Cardiovascular (CiberCV), Madrid, Spain

**Keywords:** dilated cardiomyopathy, ERS, hypertrophic cardiomyopathy, lncRNAs, *Walras*, UPR

In the published article, affiliation 14, “Ciber Cardiovascular (CiberCV), Madrid, Spain”, was omitted for authors “Alejandro Salguero and Jose Luis De La Pompa”. The correct affiliations for these authors are affiliations “13 and 14”:

“13 Intercellular Signaling in Cardiovascular Development and Disease Laboratory, Centro Nacional de Investigaciones Cardiovasculares Carlos III, Madrid, Spain”.

“14 Ciber Cardiovascular (CiberCV), Madrid, Spain”.

The omission of affiliation 14 does not affect the scientific conclusions of the article in any way. The conflict of interest statement remains unchanged.

The original article has been updated.

