# Supplementary material for: Walras modulates sex-dependent endoplasmic reticulum stress in cardiomyopathy
Source: Front Physiol. 2026 May 11;17:1740128. doi: 10.3389/fphys.2026.1740128 (PMC13199116; doi:10.3389/fphys.2026.1740128)

SUPPLEMENTARY FIGURE 1

A

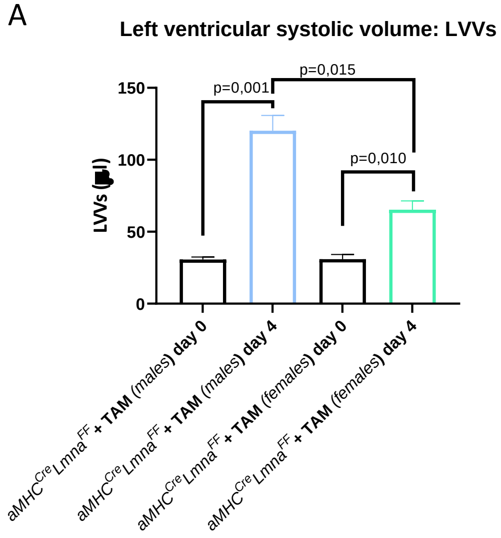

B

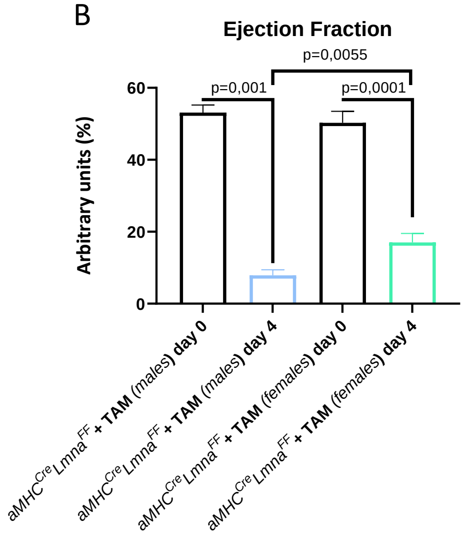

SUPPLEMENTARY FIGURE 2

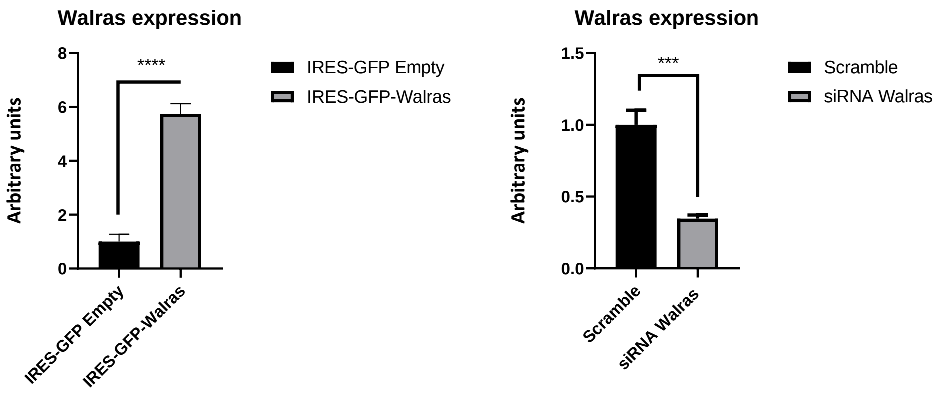

SUPPLEMENTARY FIGURE 3

Primary cardiomyocyte culture

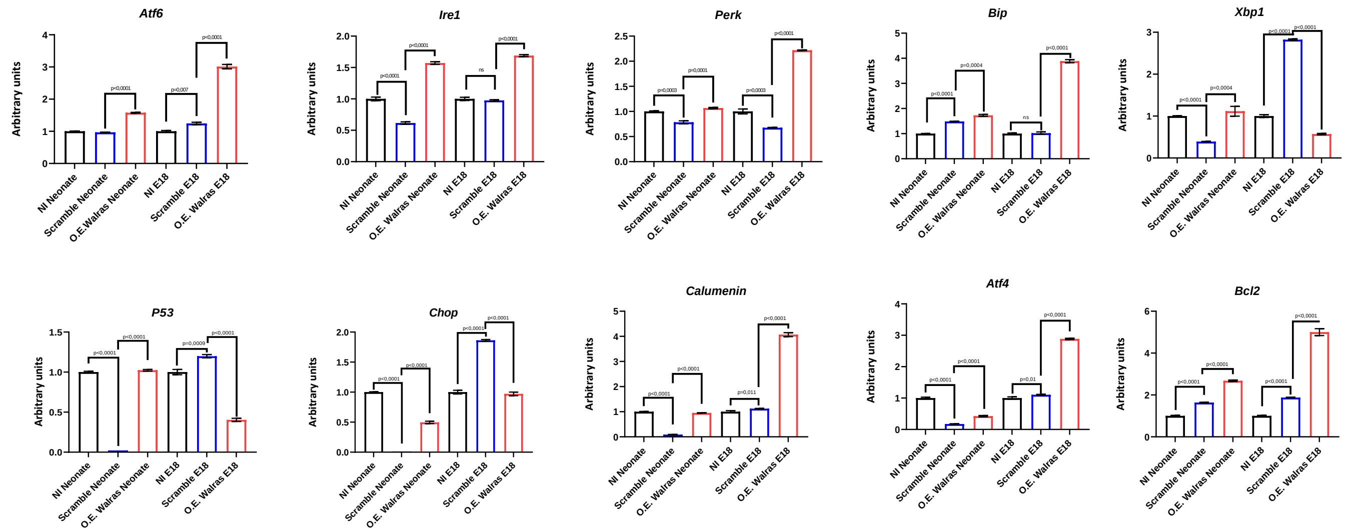

Cardiac fibroblast culture

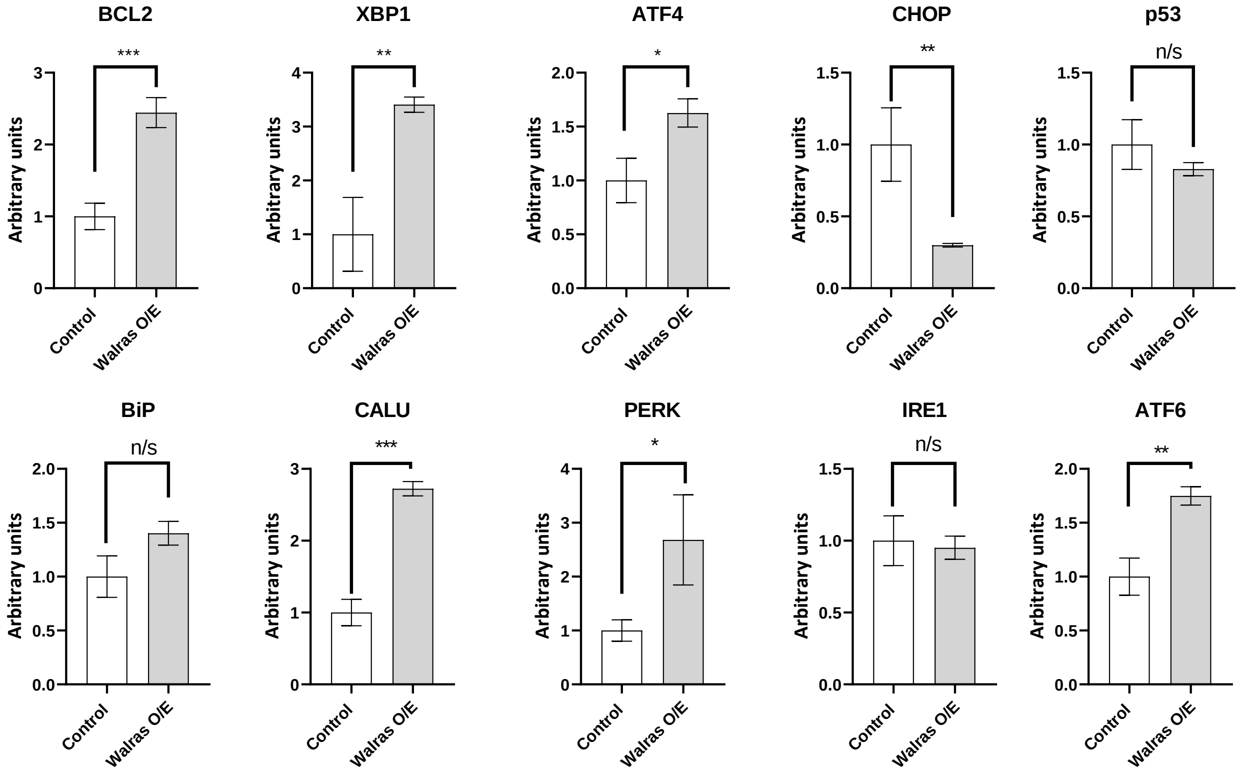

SUPPLEMENTARY FIGURE 4

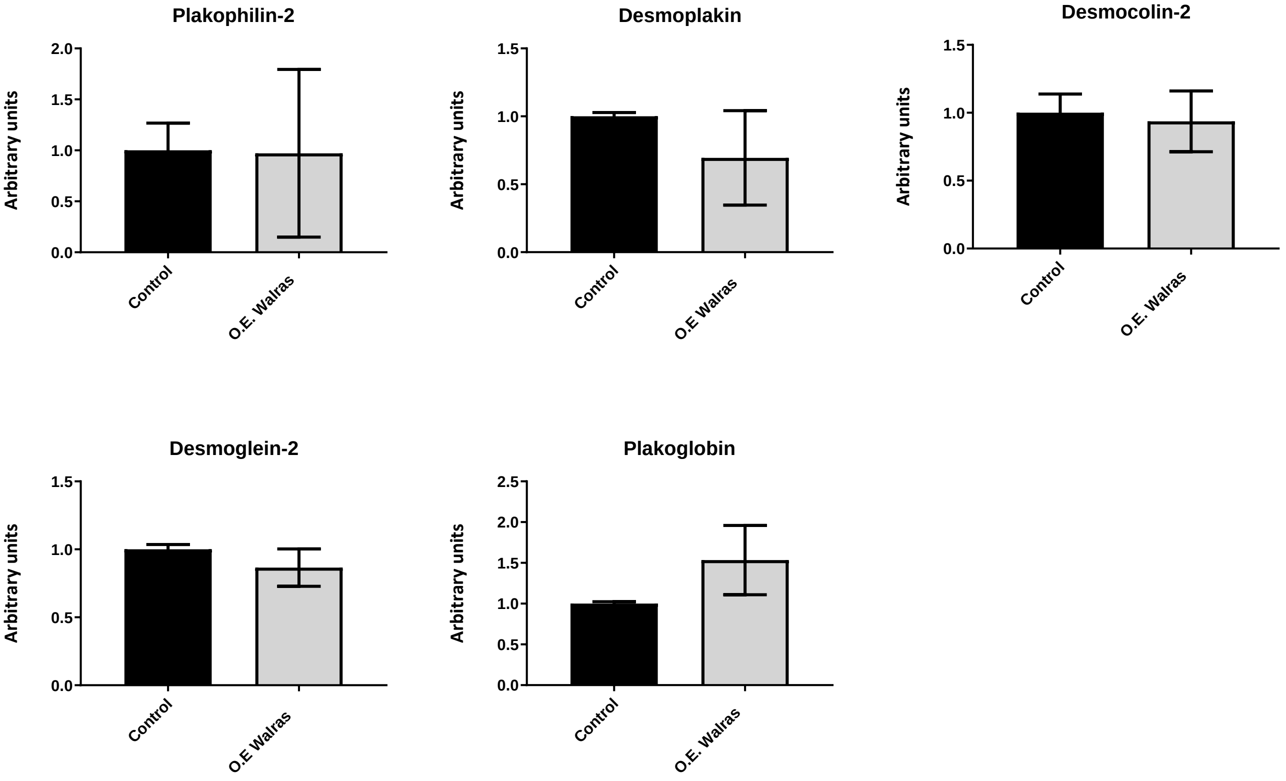

SUPPLEMENTARY FIGURE 4

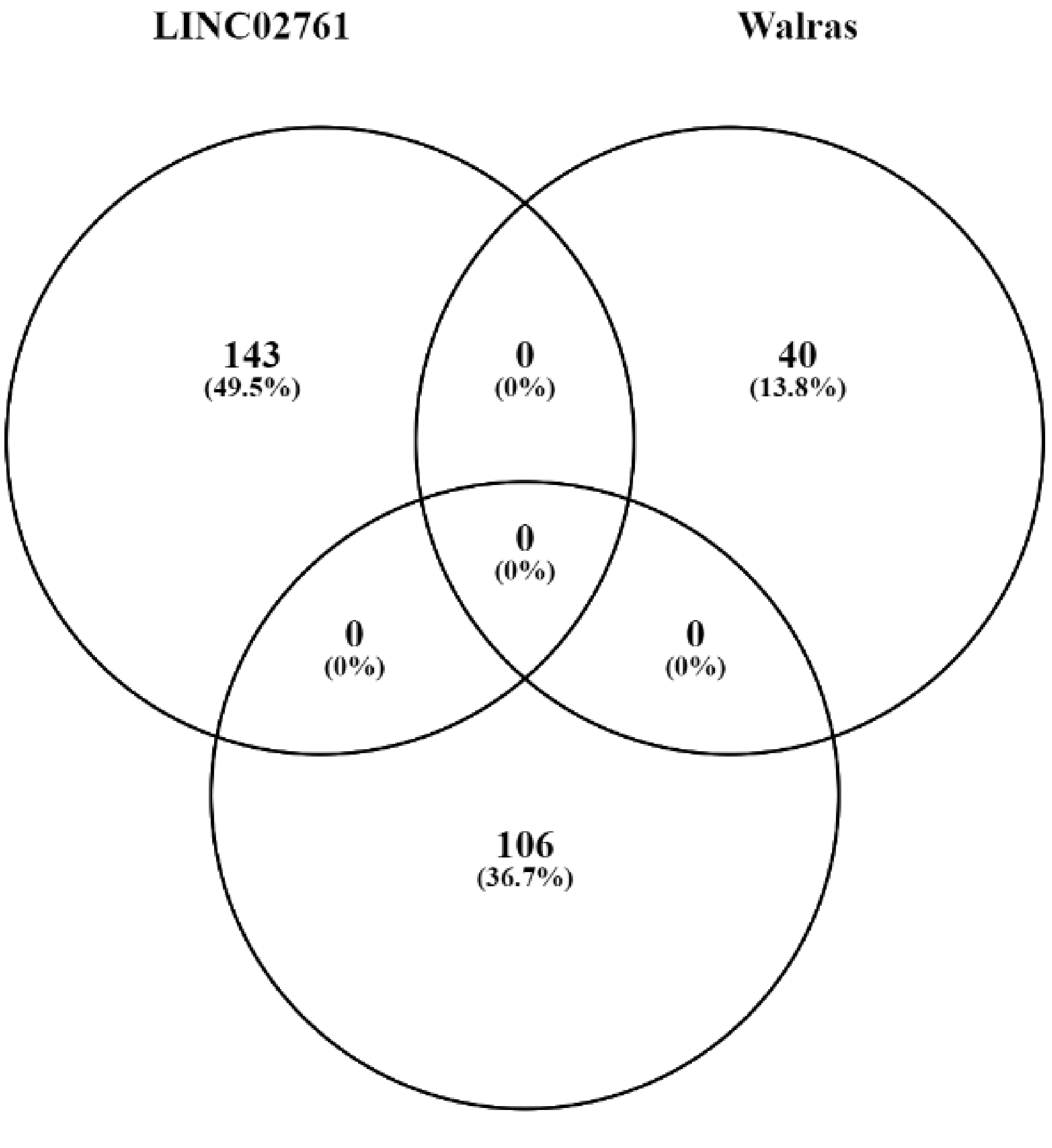

Supplement: Supplementary Data 1 — Echography heart analysis of αMHCCre LmnaFF + TAM miles and mices. Left ventricular systolic volumen and Ejection Fraction of αMHCCre LmnaFF + TAM mices at day 0 and 4 after Tamoxyfen administration (Panels A-B) (n=3 per group). Note that LVVs is increased in males compared to females whereas ejection fraction is lower in males compared to females. Three biological samples were used in each analysis. Statistical analysis: Student’s t (95% confidence interval); * p-value < 0.05; ** p-value < 0.01; *** p-value < 0.001; **** p-value < 0.0001. [file DataSheet1.pdf]
