# Supplementary material for: Walras modulates sex-dependent endoplasmic reticulum stress in cardiomyopathy
Source: Front Physiol. 2026 May 11;17:1740128. doi: 10.3389/fphys.2026.1740128 (PMC13199116; doi:10.3389/fphys.2026.1740128)
Supplement: Supplementary Figure 5 — Venny’s diagrams of LINC02761, Walras and APO02340.1. Observe that any protein is shared by LINC02761, APO02340.1 and Walras (n=3 per group). [file Table1.docx]

***Supplementary table 1: Primers Sequences***

| Gene | Sequence | Specie |
| --- | --- | --- |
| Walras/Gm44934 F | GCTACAATGGAGGGCTTCAG | Mus musculus |
| Walras/Gm44934 R | CAGCTTCTTCATGGCCTCAT | Mus musculus |
| Gm26538 F | ACASGCAAAGGATTGGTGAGC | Mus musculus |
| Gm26538 R | TTACAGAAGAGCCTGCCACA | Mus musculus |
| Walaa/Gm 45188 F | ACAGAGAATACGGGCACACC | Mus musculus |
| Walaa/Gm 45188 R | TCAGAGGAGCTTCCGAGAAA | Mus musculus |
| Walrad/Gm44653 F | GGTTCTGAAGAGGGCAAAGA | Mus musculus |
| Walrad/Gm44653 R | CCACTACCATAGGGGCTGTG | Mus musculus |
| Wallrd/2010110K18Rik F | GCTGAGACCGATGAAGTGGT | Mus musculus |
| Wallrd/2010110K18Rik R | CATCCTTGTGGCTGCCTACA | Mus musculus |
| ATF6 F | TACCACCCACAACAAGACCA | Mus musculus |
| ATF6 R | TGATGATCCCGGAGATAAGG | Mus musculus |
| IRE1 F | CGAATAGAAAAGGAGGCCTTG | Mus musculus |
| IRE1 R | CTCGGAGGAGGTCTCTCACA | Mus musculus |
| PERK F | TTCATGGAAACAACTACTCCCATA | Mus musculus |
| PERK R | TGGGGATATTTCTGAGTGAACA | Mus musculus |
| BIP F | CAGATCTTCTCCACGGCTTC | Mus musculus |
| BIP R | TTCAGCTGTCACTCGGAGAA | Mus musculus |
| ATF4 F | GAAACCTCATGGGTTCTCCA | Mus musculus |
| ATF4 R | AGAGCTCATCTGGCATGGTT | Mus musculus |
| Bcl-2 F | AGTACCTGAACCGGCATCTG | Mus musculus |
| Bcl-2 R | CAGGTATGCACCCAGAGTGA | Mus musculus |

| Caluminina F | TCGAGATAAGAACCGGGATG | Mus musculus |
| --- | --- | --- |
| Caluminina R | CTGGCTGCCCACAAATAAAT | Mus musculus |
| P53 F | GCGTAAACGCTTCGAGATGT | Mus musculus |
| P53 R | CCCCACTTTCTTGACCATTG | Mus musculus |
| Chop F | CTGCCTTTCACCTTGGAGAC | Mus musculus |
| Chop R | GGACGCAGGGTCAAGAGTAG | Mus musculus |
| Xbp1 F | TCCGCAGCACTCAGACTATG | Mus musculus |
| Xbp1 R | ACAGGGTCCAACTTGTCCAG | Mus musculus |
| LINC02761 F | CCAGAGGCTGAGTGACTTCC | Homo sapiens |
| LINC02761 R | CGCAGCGTGGTTACTGTAGA | Homo sapiens |
| AP002340.1 F | TTACAGGTGTGAGCCACAGG | Homo sapiens |
| AP002340.1 R | CCAGAGGCAACGAGAAAATGC | Homo sapiens |
| ATF6 F | TCTCGTGGCCTTCTTGTTCT | Homo sapiens |
| ATF6 R | ACCCACCTTTTCACCTGGAA | Homo sapiens |
| IRE1 F | CCTGGGGCAAGAGGTAAAGA | Homo sapiens |
| IRE1 R | AGTTTGACTGCCTACTCGCT | Homo sapiens |
| PERK F | AGACATGCTCTCTCCATCCC | Homo sapiens |
| PERK R | AGGGCTATGGGAGTTGTTGG | Homo sapiens |
